# Flexible Vertex Engineers the Controlled Assembly of Distorted Supramolecular Tetrahedral and Octahedral Cages

**DOI:** 10.34133/2022/9819343

**Published:** 2022-02-24

**Authors:** Shu-Jin Bao, Ze-Ming Xu, Tian-Chen Yu, Ying-Lin Song, Heng Wang, Zheng Niu, Xiaopeng Li, Brendan F. Abrahams, Pierre Braunstein, Jian-Ping Lang

**Affiliations:** ^1^College of Chemistry, Chemical Engineering and Materials Science, Soochow University, Suzhou 215123, China; ^2^State Key Laboratory of Organometallic Chemistry, Shanghai Institute of Organic Chemistry, Chinese Academy of Sciences, Shanghai 200032, China; ^3^School of Physical Science and Technology, Soochow University, Suzhou 215006, China; ^4^College of Chemistry and Environmental Engineering, Shenzhen University, Shenzhen 518071, China; ^5^School of Chemistry, University of Melbourne, Victoria 3010, Australia; ^6^Université de Strasbourg-CNRS, Institut de Chimie (UMR 7177 CNRS), 4 Rue Blaise Pascal CS 90032, 67081 Strasbourg, France

## Abstract

Designing and building unique cage assemblies attract increasing interest from supramolecular chemists but remain synthetically challenging. Herein, we propose the use of a flexible vertex with adjustable angles to selectively form highly distorted tetrahedral and octahedral cages, for the first time, in which the flexible vertex forms from the synergistic effect of coordination and covalent interactions. The inherent interligand angle of the vertex can be modulated by guest anions present, which allows for the fine-tuning of different cage geometries. Furthermore, the reversible structural transformation between tetrahedral and octahedral cages was achieved by anion exchange monitored by mass spectrometric technique, the smaller anions favoring tetrahedral cages, while the larger anions supporting octahedral cages. Additionally, the KBr-based cage thin films exhibited prominent enhancement of their third-order NLO responses in two or three orders of magnitude compared to those obtained for their corresponding solutions. This work not only provides a new methodology to build irregular polyhedral structures in a controlled and tunable way but also provides access to new kinds of promising functional optical materials.

## 1. Introduction

**Figure 1:**
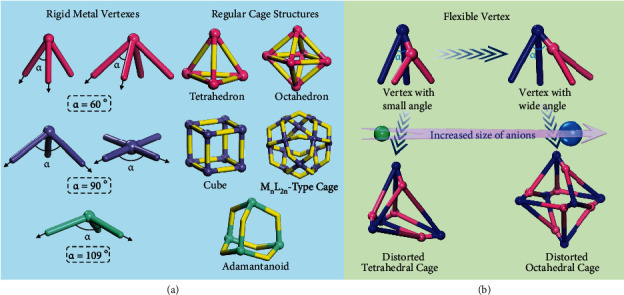
Types of metal coordination building blocks used for constructing supramolecular cage structures. (a) Previous work showing the current use of rigid metal vertexes with predetermined interligand angles (60°, 90°, and 109°), leading to regular cage structures, mainly tetrahedral, octahedral, cubic, M_n_L_2n_-type polyhedral, and adamantanoidal cages. (b) New design approach proposed in this work. Taking advantage of a type of flexible coordination vertex capable of adapting angular changes in response to the changes of the size of guest anions, the controlled construction of distorted supramolecular tetrahedral and octahedral cages can be achieved.

Over the past decades, designing and synthesizing unique supramolecular cages featuring intriguing polyhedral geometries and confined cavities have sparked great interest in supramolecular community [1–6]. In addition to their aesthetically pleasing structures, their unique physical and chemical properties resulting from specific shapes and functionalities endow them wide applications across various fields, ranging from biomedicine [7–9], catalysis [10–14], guest encapsulation [15–17], separation [18, 19] to luminescent materials [20–22]. Recent progress allows the structures of supramolecular cages to be readily designed and synthesized using highly directional coordination bonds linking metal ions with specific coordination geometries with organic linkers of different symmetries, lengths, and steric bulk [1–6, 23–26]. If the directionality of coordination bonds has critically contributed to spectacular developments in the field, access to only a limited number of interligand angles significantly hinders the development of higher structural diversity and complexity [27, 28]. Typically, the most encountered interligand angles are centered around 60°, 90°, and 109° and cannot be easily modified because they are dictated by the nature of the metal center [28]. These angles favor the formation of regular cages with tetrahedral, octahedral, cubic, M_n_L_2n_-type polyhedral, and adamantanoidal structures (Figure 1(a)) [1, 29, 30]. Consequently, being able to adjust the interligand angles at a vertex would represent a highly desirable and major step forward to efficiently expand the existing libraries of molecular cages.

However, the coordination interaction-based vertices used for the construction of supramolecular cage architectures are limited to single/double metal nodes [1, 31] and metal cluster nodes (carboxylate-containing and polyoxometalate clusters) [32–35], which are quasirigid elementary bricks that significantly restrict angular changes on vertexes. Therefore, the introduction of additional interactions, such as covalent interactions, would provide vertexes with higher flexibility and distorted characteristic and thus access to unconventional supramolecular cages [36]. In particular, if the variable angles at a flexible vertex could be tuned by an external stimulus, such as a guest molecule, the controllable construction of diverse supramolecular cage architectures would be more readily accessible [5, 37–40].

We herein report a new strategy based on a type of flexible vertex with adjustable angles to selectively construct a series of severely distorted tetrahedral and octahedral cages. The synergistic effect of both coordination and covalent interactions gives the vertex flexible and low-symmetry merits. Consistent with the possibility of the associated anion to act as a template and guide the assembly of supramolecular structures [41–45], we have now observed that the size of guest anions allowed a controllable angular modulation of a flexible vertex, facilitating the selective formation of irregular polyhedral structures (Figure 1(b)). Remarkably, the transformation between the cages containing a smaller anion and the relatively larger anions was found to be reversible, thus highlighting the impact of vertex flexibility on the structural tuning of cage assemblies.

## 2. Results

### 2.1. Design and Construction of the Flexible Vertex

**Figure 2:**
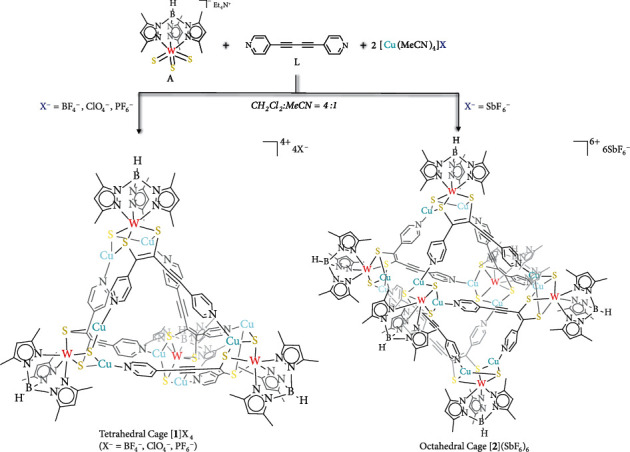
Schematic representation of the synthetic routes to the tetrahedral cage [**2**] and octahedral cage [**2**]. Treatment of the metal sulfide synthon [Et_4_N][Tp^∗^WS_3_] (**A**) (1 equiv) with the ligand 1,4-di(pyridin-4-yl)buta-1,3-diyne (**L**) (1 equiv) and [Cu(MeCN)_4_]X (X = BF_4_^−^ or ClO_4_^−^ or PF_6_^−^) (2 equiv) led to the formation of the framework of cationic distorted tetrahedral cage [**2**]. The framework of cationic distorted octahedral cage [**2**] formed from the reaction of subcomponents **A** and **L** together with [Cu(MeCN)_4_]SbF_6_ in the same stoichiometry of 1 : 1 : 2.

Considering that vertex flexibility could result from the synergistic effect of coordination and covalent interactions, the ligand 1,4-di(pyridin-4-yl)buta-1,3-diyne (**L**) (Figure 2) was chosen owing to its reactive alkyne functionality towards the metal sulfide synthon [Et_4_N][*Tp*^∗^WS_3_] (*Tp*^∗^ = hydridotris(3,5-dimethylpyrazol-1-yl)borate) (**A**) (Figure 2) to generate a tetracoordinated building block of low symmetry [36, 46]. During this reaction, **L** undergoes a significant configuration change and forms the new linker **L**^**a**^ [36, 46]. The resulting 4-coordinated building block binds with Cu(I) ions to form a new flexible vertex that can be used to construct unique supramolecular cages. In addition, the *π*-conjugated pyridyl moieties on ligand **L** can engender favorable H-bonding and anion-*π* interactions with guest anions [47–49]. Formation of these H-bonding interactions and anion-*π* interactions constitutes the driving force for regulating angular changes in the flexible vertex units.

### 2.2. Self-Assembly of the Distorted Tetrahedral Cage [***1***](BF_4_)_4_

**Figure 3:**
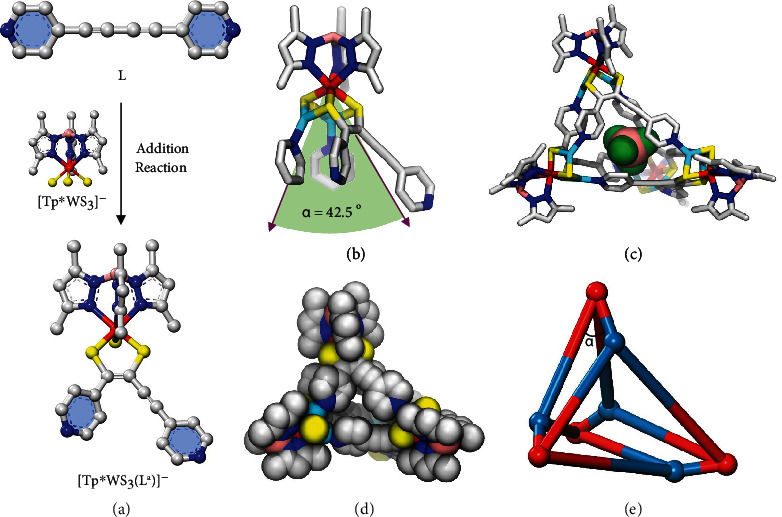
Single-crystal X-ray structure of the tetrahedral cage [**1**]. (a) View of the structure of the [*Tp*^∗^WS_3_(**L**^**a**^)]^−^ moiety from addition reaction between ligand **L** and the metal sulfide synthon [Et_4_N][*Tp*^∗^WS_3_] (**A**), producing S-C=C-S covalent interaction. (b) View of the vertex of [**1**] with a small angle of *α* being 42.5°. (c) View of the whole structure of the distorted cationic tetrahedral cage [**1**] with a space-filling model representation of the one templating BF_4_^−^ anion included. (d) View of the space-filling model representation of [**1**]. (e) Topology of [**1**]. The angle of *α* marked here corresponds to the angle of vertex in Figure 3(b). The red balls and cyan balls, respectively, stand for the *Tp*^∗^WS_3_ unit and reacted alkynyl ligand **L**^**a**^ units. Color codes: W (red), Cu (azure), S (yellow), N (blue), C (silver), B (light salmon), and F (green). In all views, all hydrogen atoms, the nonencapsulated anions, solvent molecules, and disorder are omitted for clarity.

The reaction of the metal sulfide synthon **A** (1.0 equiv), [Cu(MeCN)_4_]BF_4_ (2.0 equiv), and ligand **L** (1.0 equiv) in a mixed CH_2_Cl_2_/MeCN solvent yielded a severely distorted cationic tetrahedral cage of composition [*Tp*^∗^WS_3_Cu_2_(**L**^**a**^)]_4_(BF_4_)_4_ ([**1**](BF_4_)_4_) (Figures 2 and 3), as established by single-crystal X-ray diffraction (SCXRD). Its stoichiometry was further confirmed by electrospray ionization mass spectrometry (ESI-MS), with a peak at *m*/*z* = 1239.9343 with the correct isotopic distribution pattern for {[**1**](BF_4_)}^3+^ in MeCN, indicating that cage [**1**](BF_4_)_4_ was stable in solution (Figure S1). Furthermore, diffusion-ordered NMR spectroscopy (DOSY) experiment on [**1**](BF_4_)_4_ showed the presence of a discrete tetrahedral cage structure, with the single diffusion coefficient (*D* = 4.84 × 10^−10^m^2^ s^−1^) (Figure S10).

The SCXRD result reveals that [**1**](BF_4_)_4_ crystallizes in the triclinic space group $P\bar{1}$. As shown in Figure 3(a), one alkynyl group of **L** undergoes coupling with two S atoms on [*Tp*^∗^WS_3_]^−^ to produce covalent interactions (S−C=C−S) in a 1,2-enedithiolato [*Tp*^∗^WS_3_(**L**^**a**^)]^−^ moiety, in which three coordination sites of the W (VI) center are occupied by the *Tp*^∗^ and the octahedral coordination sphere is completed by three S atoms. This addition reaction resulted in a significant change of the geometry of **L** from linear to “boat” in the newly formed linker **L**^**a**^. The remaining terminal S atom of **A** and two S (S−C=C−S) atoms are available for binding with two Cu(I) ions, thus forming the [*Tp*^∗^WS_3_Cu_2_(**L**^**a**^)]^+^ moiety that can be regarded as a single “vertex” (Figure 3(b)). Both Cu(I) ions on the [*Tp*^∗^WS_3_Cu_2_(**L**^**a**^)]^+^ vertex further bind to the pyridine donor of **L**^**a**^ extending from other [*Tp*^∗^WS_3_Cu_2_(**L**^**a**^)]^+^ units via coordination interactions. The coexistence of coordination and covalent interactions in one connector lowers the symmetry of the vertex and renders the resulting supramolecular architectures dissymmetrical and distorted. The interligand angle *α* in the [*Tp*^∗^WS_3_Cu_2_(**L**^**a**^)]^+^ vertex is of approximately 42.5° (Figure 3(b)). The four connections extend out of the [*Tp*^∗^WS_3_Cu_2_(**L**^**a**^)]^+^ unit, but it is clear that the two short connections, involving both a pyridyl donor and acceptor, are directed towards a single [*Tp*^∗^WS_3_Cu_2_(**L**^**a**^)]^+^ unit, which further links only three other chemically similar, but crystallographically distinct [*Tp*^∗^WS_3_Cu_2_(**L**^**a**^)]^+^ units. This results in a severely distorted, dissymmetrical tetrahedral cage (Figure 3(c) and 3(d)) possessing an unprecedented topology (Figure 3(e)). This dissymmetrical character results in a chiral tetrahedral structure (Figure S22), but the enantiomeric cages crystallize in a 1 : 1 stoichiometry (Figure S23) to form a racemic conglomerate.

The internal BF_4_^−^ anion in the tetrahedral cage [**1**] fits snugly within the hollow (Figure 3(c)), which suggests that the internal anion plays an important templating role in the formation of the tetrahedral cage. It is found to associate with the tetrahedral host in which the F atoms interact with the close H atoms of adjacent pyridine rings on the **L**^**a**^ linker, forming H-bonding interactions, with the primary C−H···F distances in the range of 2.61–2.87 Å (Figure S24A). Furthermore, it is engaged in typical anion-*π* contacts in which the F atoms interact with the C atoms on the pyridine rings, establishing the directional F···C_pyridine_ contacts in the range of 3.07–3.45 Å (Figure S24B). The above close-contact analysis of cage [**1**] (BF_4_)_4_ shows that the framework of tetrahedral host is well stabilized by H-bonding and F-*π* interactions between the pyridyl moieties and the encapsulated BF_4_^−^.

### 2.3. Impact of Guest Anions on the Angular Changes in a Flexible Vertex

The host-guest interactions between the internal BF_4_^−^ and the tetrahedral host [**1**] indicate that this guest anion acts as a competent template in the self-assembly process and the larger anions may generate similar interactions with the [*Tp*^∗^WS_3_Cu_2_(**L**^**a**^)]^+^ units and influence the angle *α* at the node, thereby possibly realizing a fine-tuning of supramolecular cage geometries. To verify this assumption, three other anions with increasing volume, oxyanion perchlorate (ClO_4_^−^), hexafluorophosphate (PF_6_^−^) and hexafluoroantimonate (SbF_6_^−^) were adopted. Using similar reaction conditions as for [**1**](BF_4_)_4_, two isostructural tetrahedral assemblies [**1**](ClO_4_)_4_ (Figure S25A) and [**1**](PF_6_)_4_ (Figure S25B) were obtained, respectively, with the value for the angle *α* in the vertex approximately equal to that in the counterpart in [**1**](BF_4_)_4_ (Figure S26). Each tetrahedral cage encapsulates one ClO_4_^−^ or one PF_6_^−^ within its cavity, and similar host-guest interactions are also observed as described in [**1**](BF_4_)_4_ (Figure S27 and Figure S28). Interestingly, when the larger anion SbF_6_^−^ was employed, a cationic octahedral assembly [*Tp*^∗^WS_3_Cu_2_(**L**^**a**^)]_6_(SbF_6_)_6_ ([**2**](SbF_6_)_6_) (Figure 2 and Figure S30) was isolated, which crystallized in the trigonal space group $R\overset{¯}{3}$. The above solid compounds had good thermal stability (Figure S31) confirmed by the thermogravimetric analyses (TGA), and the octahedral compound [**2**](SbF_6_)_6_ exhibited higher thermal stability than the tetrahedral compounds.

**Figure 4:**
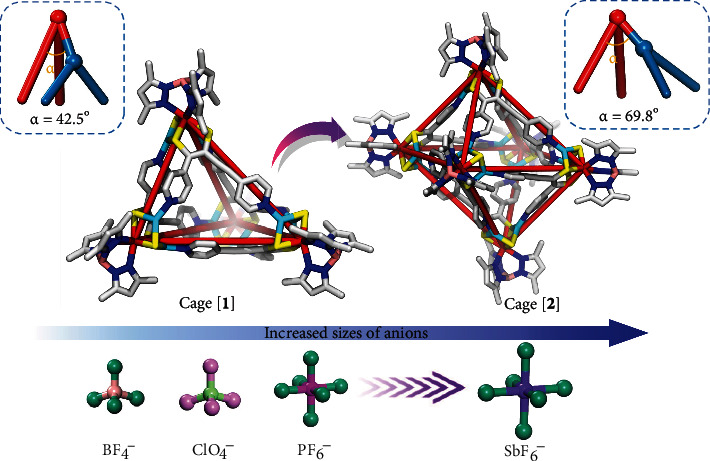
Illustration of the controllable formation of tetrahedral cage [**1**] and octahedral cage [**2**] by regulating the angle at a flexible vertex by the change of the anion size. The smaller anions including BF_4_^−^, ClO_4_^−^, and PF_6_^−^ favor to the formation of the small-angle vertex with approximately 42.5°, generating highly distorted tetrahedral cage [**1**]. When the size of the anion further increases from PF_6_^−^ to SbF_6_^−^, the angle of the flexible vertex significantly opens to 69.8°, thus causing the structural transition from the tetrahedral cage [**1**] to distorted octahedral cage [**2**].

In the octahedral cage [**2**], although the [*Tp*^∗^WS_3_Cu_2_(**L**^**a**^)]^+^ unit serves as a 4-connector and as a structural vertex, the angle *α* is dramatically increased to 69.8° (Figure 4 and Figure S30C). This nicely validates our speculation that the [*Tp*^∗^WS_3_Cu_2_(**L**^**a**^)]^+^ unit functions as a versatile vertex that is able to stretch its interligand angles ranging from 42.5° to 69.8° in response to the large SbF_6_^−^ anions. The vertexes containing the angle *α* being 69.8° allow to be splayed apart from each other permitting connections to four rather than three equivalent units, thus leading to a low symmetry cage of distorted octahedral geometry (Figure S30D). This exemplifies the real possibility of controlling the assembled structure through adjusting the angle at a flexible vertex.

Inspecting the cavity of the octahedral cage [**2**] shows that there is one pair of SbF_6_^−^ anions (Figure S30A). Each Sb(V) center is located just off a 3-fold axis that passes through the cage. In addition to the positional disorder, the internal anions exhibit considerable orientational disorder (only one orientation is shown in Figure S30A, Figure S32, and Figure S33). Despite this disorder, it is apparent that the F atoms establish a rather close contact with the aromatic surfaces. Both encapsulated SbF_6_^−^ anions develop hydrogen bonding contacts with the pyridyl H atoms, with the primary C−H···F distances being 2.08–2.97 Å (Figure S33A). In addition, the C atoms of the linker **L**^**a**^ also establish directional F···C_pyridine_ contacts with the F atoms of the SbF_6_^−^ anions with the primary distances being 3.12–3.60 Å (Figure S33B). Interestingly, the pyridyl group of the linker **L**^**a**^ in [**2**](SbF_6_)_6_ is oriented in such a way that the hydrogen atoms of the pyridyl group are pointing inwards in a configuration that results in the C−H···F contacts. Examination of each structure suggests that the angle at the vertex could be adjusted in a controlled manner, adapting to the changes of the size of guest anions. When the size of guest anions increases from BF_4_^−^, ClO_4_^−^, PF_6_^−^ to SbF_6_^−^, the angle in the metal vertex drastically expands from 42.5° to 69.8°, resulting in the structural transition from the tetrahedral cage [**1**] to the octahedral cage [**2**] (Figure 4).

### 2.4. Reversible Structural Transformation between [***1***] and [***2***]

**Figure 5:**
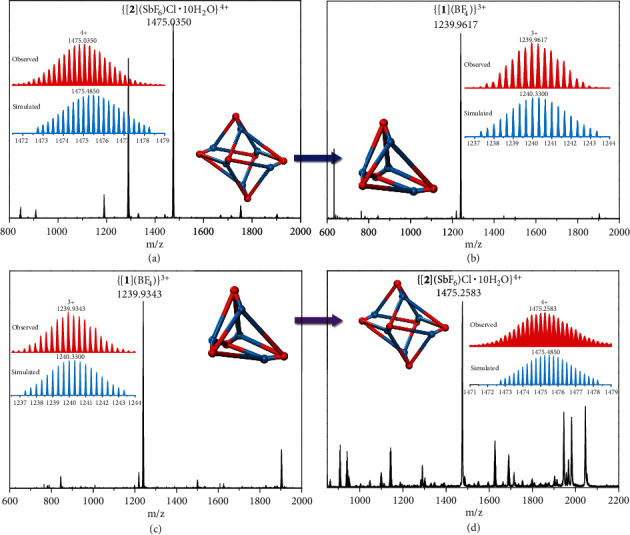
Monitoring the structural transformations between tetrahedral cage [**1**] and octahedral cage [**2**]. (a) ESI-MS spectrum of the pure octahedral compound [**2**](SbF_6_)_6_ in MeCN before addition of (^n^Bu_4_N)(BF_4_). (b) ESI-MS spectrum after addition of excess (^n^Bu_4_N)(BF_4_) successfully monitoring the appearance of the tetrahedral cage [**1**] at *m*/*z* 1239.9617. (c) ESI-MS spectrum of the pure tetrahedral compound [**1**](BF_4_)_4_ in MeCN.(d) ESI-MS spectrum after addition of HSbF_6_ and NEt_3_ successfully monitoring the appearance of the octahedral cage [**2**] at *m*/*z* 1475.2583.

The anion-dependent formation of the tetrahedral and octahedral assemblies encouraged us to explore their interconversion starting from the respective pure entities. We speculated that a change of the anion might result in a unique reversible conversion between tetrahedral [**1**] and octahedral cages [**2**]. After dissolution of the octahedral cage compound [**2**](SbF_6_)_6_ in MeCN (Figure 5(a)), an excess of (^n^Bu_4_N)(BF_4_) was added to the MeCN solution stirring for 8 h at ambient temperature. ESI-MS analysis revealed a new peak at *m*/*z* 1239.9617, assigned to the tetrahedral cage {[**1**](BF_4_)}^3+^ with the correct isotopic distribution (Figure 5(b)). This demonstrated that introduction of small BF_4_^−^ anions to a solution of the octahedral cage compound [**2**] induces its transformation to the tetrahedral cage [**1**]. However, when we introduced excess (Et_4_N)(SbF_6_) to the tetrahedral [**1**](BF_4_)_4_ system, no any octahedral cage [**2**] signal (Figure S34) was observed, indicating no cage transformation took place. Intriguingly, upon addition of HSbF_6_ and NEt_3_ to a solution of [**1**](BF_4_)_4_, we did observe the structural transformation of the tetrahedral cage [**1**] to the octahedral cage [**2**] by ESI-MS. One signal at *m*/*z* 1475.2583 can be assigned to be the octahedral cage {[**2**](SbF_6_)(Cl)·10H_2_O}^4+^ (Figures 5(c) and 5(d)). The results demonstrate anion-directed reversible transformation between the tetrahedral cage [**1**] and the octahedral cage [**2**] and further validate the concept of flexible building block where the angle at the vertex is capable of adjusting to the nature of the anion present, to give the preferred cage structure.

### 2.5. Investigation of Third-Order Nonlinear Optical (NLO) Properties

**Figure 6:**
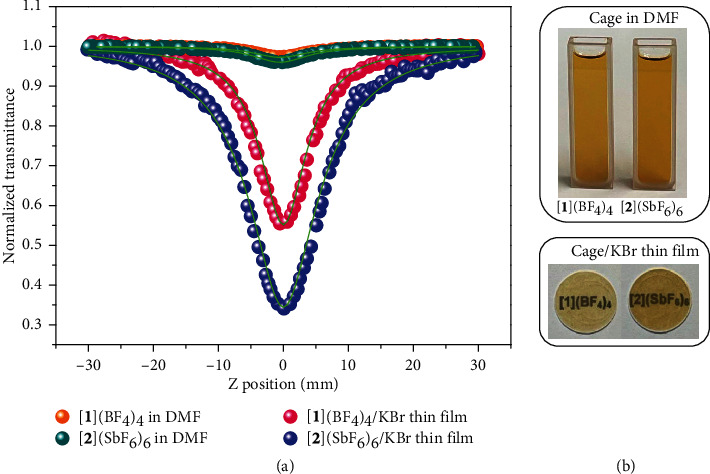
Third-order NLO properties of the KBr-based thin films of [**1**](BF_4_)_4_ and [**2**](SbF_6_)_6_ and their DMF solutions. The orange, dark cyan, pink, and blue spheres represent the experimental data of [**1**](BF_4_)_4_ in DMF, [**2**](SbF_6_)_6_ in DMF, [**1**](BF_4_)_4_/KBr thin film, and [**2**](SbF_6_)_6_/KBr thin film, respectively, and the green solid curves represent the theoretical fit by Bahae et al.'s theory [52]. The insets in (b) are photo of the samples of [**1**](BF_4_)_4_ and [**2**](SbF_6_)_6_ in DMF and KBr-based cage thin films.

The rigid skeletons of the above cage assemblies hold multiple heavy metal atoms and highly *π*-conjugated organic linkers, which inspired us to explore their third-order nonlinear optical (NLO) properties [36, 50]. The third-order NLO properties of KBr-based thin films of **A**, [**1**](BF_4_)_4_, and [**2**](SbF_6_)_6_ prepared by a table pressing method and their DMF solutions (1.38 × 10^−4^ mol/L) were measured, respectively, by a typical open aperture Z-scan technique at 532 nm with a nanosecond pulsed (4 ns) laser under the similar incident pulse energy. Both cage compounds had deep valleys at zero point (focal point of lens), indicating an interesting reverse saturable absorption (RSA) response [51] either in solution or in an aggregated state of the KBr-based thin films (Figure 6). But for pure DMF solvent, pure KBr thin film, **A** in DMF, and **A**/KBr thin film, they show no detectable absorption signals (Figures S37-S40). Thus, the third-order NLO responses could result from the tetrahedral or octahedral cages. Both [**1**](BF_4_)_4_ and [**2**](SbF_6_)_6_ in DMF showed a very weak RSA signal with the effective nonlinear absorption coefficient *β* [52] of 1.1 × 10^−11^ m/W and 1.8 × 10^−11^ m/W, respectively. However, the NLO responses of their KBr-based thin films were sharply enhanced, with their corresponding *β* values being 7.0 × 10^−9^ m/W for [**1**](BF_4_)_4_/KBr thin film and 2.1 × 10^−8^ m/W for [**2**](SbF_6_)_6_/KBr thin film, which are 636 and 1167 times larger than those of their corresponding solutions (Table S2) and better than that of [(C_4_H_9_)_4_]_2_[Cu(C_3_S_5_)_2_]-doped polymethylmethacrylate (PMMA) thin film [53]. Similar Z-scan results for [**1**](ClO_4_)_4_ (Figure S41 and Table S2) and [**1**](PF_6_)_4_ (Figure S42 and Table S2) were also obtained. Such an outstanding enhancement of the NLO performances could be due to doping concentrations in the KBr films being much larger than those of solution. The difference between the KBr-based tetrahedral and octahedral films may be ascribed to the increase of W/Cu/S cluster cores and the structural expansion from a tetrahedral cage to an octahedral cage. In addition, the third-order NLO response could be gradually improved by increasing the concentrations of [**1**](BF_4_)_4_ and [**2**](SbF_6_)_6_ in DMF by 1.5 times (2.07 × 10^−4^ mol/L) and 2 times (2.76 × 10^−4^ mol/L) (Figure S43, Figure S44, and Table S2).

## 3. Discussion

We have demonstrated that taking advantage of a flexible vertex with adjustable angles represents a feasible and promising strategy for creating new cage assemblies. Using this approach, two kinds of cages, highly distorted tetrahedral and distorted octahedral cages, have been constructed. Relatively small guest anions (BF_4_^−^, ClO_4_^−^, and PF_6_^−^) favor the formation of the small-angle vertex and trigger the generation of the highly distorted tetrahedral cages, whereas the larger SbF_6_^−^ anions increase the angle of the vertex and thus lead to the formation of distorted octahedral cages. The structures of these two types of supramolecular cages with host-guest interactions were demonstrated by detailed X-ray crystallography studies. Furthermore, the reversible topological transformation between these cages induced by the presence of different anions was demonstrated by ESI-MS technique. Finally, a significant amplification of the third-order NLO responses of both types of cages was realized by engineering them in KBr-based thin films, when compared to those of their solutions. This work provides a readily accessible access to topologically irregular polyhedral edifices, demonstrates anion-triggered reversible conversion between tetrahedral and octahedral cages, and paves the way for the development of new optical components.

## 4. Materials and Methods

### 4.1. Chemicals and Reagents

All reagents and solvents were purchased from commercial sources and used as supplied unless otherwise mentioned. The starting materials [Et_4_N][*Tp*^∗^WS_3_] (**A**) (*Tp*^∗^ = hydridotris(3,5-dimethylpyrazol-1-yl)borate) [54] and the ligand 1,4-di(pyridin-4-yl)buta-1,3-diyne (**L**) [55] were prepared by literature methods.

### 4.2. Physical Characterizations

Elemental analyses (C, H, and N) were performed on a Carlo-Erba CHNO-S microanalyzer. Fourier-transform infrared (IR) spectra of the solid samples (KBr tablets) in the range 400-4000 cm^−1^ were recorded on a Varian 1000 spectrometer. Thermogravimetric analyses (TGA) were performed on a Mettler Toledo Star System under a nitrogen atmosphere at a heating rate of 10°C min^−1^. UV-Vis spectra were recorded on a Varian Cary-50 UV-Vis spectrophotometer. The solid samples used for elemental analysis and TGA analysis were dried overnight at 80°C in a vacuum oven for removing solvent molecules. ^1^H NMR spectra and ^13^C NMR spectra of the ligand **L** were recorded on BRUKER AVANCE III HD (400 MHz) at room temperature and referenced to the residual protonated solvent for NMR spectra. ^1^H NMR spectra, ^1^H-^1^H COSY spectra, and ^1^H DOSY spectra of [**1**](BF_4_)_4_, [**1**](ClO_4_)_4_, [**1**](PF_6_)_4_, and [**2**](SbF_6_)_6_ were recorded on a Varian UNITY plus-600 spectrometer at room temperature and referenced to the residual protonated solvent for NMR spectra. Proton chemical shift *δ*H = 7.26 (CDCl_3_) and *δ* H = 1.94 (CD_3_CN) were reported relative to the solvent residual peak. ESI-TOF MS spectra of [**1**](BF_4_)_4_, [**1**](ClO_4_)_4_, [**1**](PF_6_)_4_, and [**2**](SbF_6_)_6_ were recorded on a Bruker micrOTOF-Q III mass spectrometer.

### 4.3. Synthesis of 1,4-Di(pyridin-4-yl)buta-1,3-diyne (L)

According to the literature procedure [55], ligand **L** was synthesized as follows. A suspension of copper (I) iodide (250 mg, 1.25 mmol), nickel (II) chloride hexahydrate (300 mg, 1.25 mmol), and tetramethylethylenediamine (TMEDA) (0.75 mL, 5.0 mmol) in 120 mL of anhydrous THF was stirred under an inert atmosphere (N_2_) for 10 min. Then, 4-ethynylpyridine (5000 mg, 50.0 mmol) was added, and the mixture was stirred at room temperature over a period of 4 h while air was bubbled through the mixture. After evaporation of the solvent, the resulting residue was chromatographed on silica gel (petroleum ether : ethyl acetate = 10 : 1) to give a colorless solid **L**: 204 mg (99%).

### 4.4. Synthesis of [***1***](BF_4_)_4_

A CH_2_Cl_2_/MeCN (40 mL/10 mL) solution of [Et_4_N][*Tp*^∗^WS_3_] (**A**) (0.071 g, 0.10 mmol), [Cu(MeCN)_4_]BF_4_ (0.063 g, 0.20 mmol), and **L** (0.020 g, 0.10 mmol) was stirred for 6 h under ambient temperature. Then, the solution was filtered, and diethyl ether was carefully layered onto the filtrate to generate red crystals in about one week. The product was isolated and characterized as indicated in the Supplementary Materials.

### 4.5. Synthesis of [***1***](ClO_4_)_4_

The synthesis method was consistent with that of compound [**1**](BF_4_)_4_. Compound [**1**](ClO_4_)_4_ was obtained by substituting [Cu(MeCN)_4_]BF_4_ (0.063 g, 0.20 mmol) for [Cu(MeCN)_4_] ClO_4_ (0.066 g, 0.20 mmol). The product was isolated and characterized as indicated in the Supplementary Materials.

### 4.6. Synthesis of [***1***](PF_6_)_4_

The synthesis method was similar to that for [**1**](BF_4_)_4_. Compound [**1**](PF_6_)_4_ was obtained by substituting [Cu(MeCN)_4_]BF_4_ (0.063 g, 0.20 mmol) for [Cu(MeCN)_4_] PF_6_ (0.075 g, 0.20 mmol). The product was isolated and characterized as indicated in the Supplementary Materials.

### 4.7. Synthesis of [***2***](SbF_6_)_6_

The synthesis method was similar to that of compound [**1**](BF_4_)_4_. Compound [**2**](SbF_6_)_6_ was obtained by substituting [Cu(MeCN)_4_]BF_4_ (0.063 g, 0.20 mmol) for [Cu(MeCN)_4_] SbF_6_ (0.093 g, 0.20 mmol). The product was isolated and characterized as indicated in the Supplementary Materials.

### 4.8. Structural Transformation from the Octahedral Cage [***2***] to the Tetrahedral Cage [***1***]

Upon dissolution of crystals of [**2**](SbF_6_)_6_ (0.006 g, 8.7 × 10^−4^ mmol) in 2 mL MeCN, an excess of (^n^Bu_4_N)(BF_4_) (0.003 g, 9.4 × 10^−3^ mmol) was added to the MeCN solution of [**2**](SbF_6_)_6_ and stirring was maintained for 8 h at ambient temperature. The solution was then analyzed by ESI-MS, and a new signal was observed at *m*/*z* 1239.9617 with the correct isotopic distribution patterns for the tetrahedral cage {[**1**](BF_4_)}^3+^. The results suggested that a structural transformation from the octahedral cage [**2**] to the tetrahedral cage [**1**] has resulted from the introduction of excess BF_4_^−^ anions.

### 4.9. Structural Transformation from the Tetrahedral Cage [***1***] to the Octahedral Cage [***2***]

Upon dissolution of crystals of [**1**](BF_4_)_4_ (0.004 g, 1.0 × 10^−3^ mmol) in 0.5 mL MeCN, HSbF_6_ (0.004 g, 0.016 mmol) in 1.5 mL MeCN was added to the MeCN solution of [**1**](BF_4_)_4_ and stirring was maintained for 2 h at ambient temperature. Then, NEt_3_ (0.002 g, 0.020 mmol) in 0.5 mL MeCN was added to the reaction mixture which was further stirred for 8 h, and then analyzed by ESI-MS. A new signal was observed at *m*/*z* 1475.2583, which was assigned to the octahedral cage {[**2**](SbF_6_)(Cl)·10H_2_O}^4+^. These experiments indicated that the structural transformation from the tetrahedral cage [**1**] to the octahedral cage [**2**] has been triggered by the larger anion (SbF_6_^−^).

### 4.10. Preparation of [***1***](BF_4_)_4_/KBr Thin Film

Transparent and uniform wafers suitable for third-order NLO tests were obtained by KBr pressed-disk technique. Firstly, [**1**](BF_4_)_4_ (0.002 g, 0.0005 mmol) was ground into a fine powder in an agate mortar. Then, dry pure KBr (AR grade) (0.500 g) was added to the agate mortar and ground with the powder of [**1**](BF_4_)_4_, giving a particle size of ca. 2 *μ*m (200 mesh). Finally, ca. 0.025 g of the mixture was evenly spread in a clean mold. An automatic powder press of 8 tons was maintained for 20 to 30 s to give a transparent sheet that was used for the NLO test. The diameter and thickness of the transparent sheet were 13 mm and 0.12 mm, respectively.

### 4.11. Preparation of [***1***](ClO_4_)_4_/KBr Thin Film

The preparation method of [**1**](ClO_4_)_4_/KBr thin film was similar to that used for the preparation of [**1**](BF_4_)_4_/KBr thin film. The [**1**](ClO_4_)_4_/KBr thin film was obtained by substituting [**1**](BF_4_)_4_ (0.002 g, 0.0005 mmol) for [**1**](ClO_4_)_4_ (0.002 g, 0.0005 mmol). The diameter and thickness of the transparent sheet were 13 mm and 0.12 mm, respectively.

### 4.12. Preparation of [***1***](PF_6_)_4_/KBr Thin Film

The preparation method of [**1**](PF_6_)_4_/KBr thin film was similar to that used for the preparation of [**1**](BF_4_)_4_/KBr thin film. The [**1**](PF_6_)_4_/KBr thin film was obtained by substituting [**1**](BF_4_)_4_ (0.002 g, 0.0005 mmol) for [**1**](PF_6_)_4_ (0.002 g, 0.0005 mmol). The diameter and thickness of the transparent sheet were 13 mm and 0.12 mm, respectively.

### 4.13. Preparation of [***2***](SbF_6_)_6_/KBr Thin Film

The preparation method of [**2**](SbF_6_)_6_/KBr thin film was similar to that used for the preparation of [**1**](BF_4_)_4_/KBr thin film. The [**2**](SbF_6_)_6_/KBr thin film was obtained by substituting [**1**](BF_4_)_4_ (0.002 g, 0.0005 mmol) for [**2**](SbF_6_)_6_ (0.003 g, 0.0005 mmol). The diameter and thickness of the transparent sheet were 13 mm and 0.12 mm, respectively.

### 4.14. Preparation of A/KBr Thin Film

The preparation method of **A**/KBr thin film was similar to that used for the preparation of [**1**](BF_4_)_4_/KBr thin film. The **A**/KBr thin film was obtained by substituting [**1**](BF_4_)_4_ (0.002 g, 0.0005 mmol) for **A** (0.001 g, 0.0005 mmol). The diameter and thickness of the transparent sheet were 13 mm and 0.12 mm, respectively.

### 4.15. Third-Order NLO Measurements

The nanosecond Z-scan technique and a linear polarized laser light (*λ* = 532 nm; repetition rate = 10 Hz; width = 4 ns) generated from a frequency-doubled, mode-locked, Q-switched Nd: YAG laser were applied to measure their third-order NLO performance. All samples were mounted on a computer-controlled translation stage that shifted each sample along the *z*-axis, and all measurements were conducted at room temperature. The nonlinear absorption data for all samples were collected using the Z-scan technique with open-aperture settings. For the KBr-based cage thin films, the incident pulse energy of 21 *μ*j was employed. For the solution samples, the higher incident pulse energy of 28 *μ*j was employed. A valley at the zero point indicated the reverse saturable absorption (RSA) response, and the depth of the valley symbolizes the strength of RSA signal. All experimental data were numerically fitted according to Bahae et al.'s theory, and the obtained effective nonlinear absorption coefficient *β* value are listed in Table S2.

## Data Availability

All data that support the findings of this study are available from the corresponding author upon reasonable request.

## Supplementary Materials

Supplementary Materials Fig. S1: experimental (top) and theoretical (bottom) ESI-TOF-MS spectra of {[**1**](BF_4_)}^3+^ in [**1**](BF_4_)_4_. Fig. S2: experimental (top) and theoretical (bottom) ESI-TOF-MS spectra of {[**1**](ClO_4_)}^3+^ in [**1**](ClO_4_)_4_. Fig. S3: experimental (top) and theoretical (bottom) ESI-TOF-MS spectra of {[**1**](PF_6_)}^3+^ in [**1**](PF_6_)_4_. Fig. S4: experimental (top) and theoretical (bottom) ESI-TOF-MS spectra of {[**1**](PF_6_)**2**}^2+^ in [**1**](PF_6_)_4_. Fig. S5: experimental (top) and theoretical (bottom) ESI-TOF-MS spectra of {[**2**](SbF_6_)(Cl)·10H_2_O}^4+^ in [**2**](SbF_6_)_6_. Fig. S6: ^1^H NMR spectrum of ligand **L** (400 MHz, 298 K, CDCl_3_, ppm). Fig. S7: ^13^C NMR spectrum of ligand **L** (101 MHz, 298 K, CDCl_3_, ppm). Fig. S8: ^1^H NMR spectrum of [**1**](BF_4_)_4_ (600 MHz, 298 K, CD_3_CN, ppm). Fig. S9: ^1^H-^1^H COSY NMR spectrum of [**1**](BF_4_)_4_ (600 MHz, 298 K, CD_3_CN, ppm). Fig. S10: ^1^H DOSY NMR spectrum of [**1**](BF_4_)_4_ (600 MHz, 298 K, CD_3_CN, ppm). Fig. S11: ^1^H NMR spectrum of [**1**](ClO_4_)_4_ (600 MHz, 298 K, CD_3_CN, ppm). Fig. S12: ^1^H-^1^H COSY NMR spectrum of [**1**](ClO_4_)_4_ (600 MHz, 298 K, CD_3_CN, ppm). Fig. S13: ^1^H DOSY NMR spectrum of [**1**](ClO_4_)_4_ (600 MHz, 298 K, CD_3_CN, ppm). Fig. S14: ^1^H NMR spectrum of [**1**](PF_6_)_4_ (600 MHz, 298 K, CD_3_CN, ppm). Fig. S15: ^1^H-^1^H COSY NMR spectrum of [**1**](PF_6_)_4_ (600 MHz, 298 K, CD_3_CN, ppm). Fig. S16: ^1^H DOSY NMR spectrum of [**1**](PF_6_)_4_ (600 MHz, 298 K, CD_3_CN, ppm). Fig. S17: ^1^H NMR spectrum of [**2**](SbF_6_)_6_ (600 MHz, 298 K, CD_3_CN, ppm). Fig. S18: ^1^H-^1^H COSY NMR spectrum of [**2**](SbF_6_)_6_ (600 MHz, 298 K, CD_3_CN, ppm). Fig. S19: ^1^H DOSY NMR spectrum of [**2**](SbF_6_)_6_ (600 MHz, 298 K, CD_3_CN, ppm). Fig. S20: a representation of single vertex (the [*Tp*^∗^WS_3_Cu_2_(**L^a^**)]^+^ unit) consisting of the C_3_-symmetry *Tp*^∗^WS_3_ moiety, two Cu(I) ions and one linker La in tetrahedral cage [**1**]. Fig. S21: view of the structure of the tetrahedral cage [**1**](BF_4_)_4_ showing the inclusion of one BF_4_^–^ with a space filling representation within the cavity as well as three external BF_4_^–^ anions. Fig. S22: one pair of enantiomers of the tetrahedral cage of [**1**]. Fig. S23: one pair of enantiomers packing in one unit cell of [**1**](BF_4_)_4_, forming a racemic crystal. Fig. S24: the host-guest interactions between the internal BF_4_^−^ and the tetrahedral host [**1**] in [**1**](BF_4_)_4_. (A) The hydrogen bonding interactions. (B) The F···*π* interactions. Fig. S25: single-crystal X-ray structure of the tetrahedral cages [**1**](ClO_4_)_4_ and [**1**](PF_6_)_4_. Fig. S26: the topology of three isostructural tetrahedral assemblies [**1**](BF_4_)_4_, [**1**](ClO_4_)_4_, and [**1**](PF_6_)_4_, showing the angles of *α* in vertex being 42.51°, 42.26°, and 43.60°, respectively. Fig. S27: the host-guest interactions between the internal ClO_4_^–^ and the tetrahedral host [**1**] in [**1**](ClO_4_)_4_. (A) The hydrogen bonding interactions. (B) The O···*π* interactions. Fig. S28: the host-guest interactions between the internal PF_6_^–^ and the tetrahedral host [**1**] in [**1**](PF_6_)_4_. (A) The hydrogen bonding interactions. (B) The F···*π* interactions. Fig. S29: view of the asymmetric unit of [**2**](SbF_6_)_6_. Fig. S30: single-crystal X-ray structure of octahedral cage [**2**]. Fig. S31: the TGA curves of [**1**](BF_4_)_4_, [**1**](ClO_4_)_4_, [**1**](PF_6_)_4_, and [**2**](SbF_6_)_6_ in a N_2_ atmosphere from room temperature to 800°C. Fig. S32: view of the structure of the octahedral cage [**2**](SbF_6_)_6_ showing the inclusion of two SbF_6_^–^ anions with a space filling representation within the cavity as well as four external SbF_6_^–^ anions. Fig. S33: the host-guest interactions between the internal SbF_6_^–^ and the octahedral host [**2**] in [**2**](SbF_6_)_6_. (A) The hydrogen bonding interactions. (B) The F···*π* interactions. Fig. S34: the ESI-MS spectrum after addition of excess (Et_4_N)(SbF_6_) to the MeCN solution of the tetrahedral compound [**1**](BF_4_)_4_, showing no any octahedral cage signal. Fig. S35: normalized UV-Vis absorption spectra of A (8.71 × 10^−5^ M), [**1**](BF_4_)_4_ (1.53 × 10^−5^ M), [**1**](ClO_4_)_4_ (1.73 × 10^−5^ M), [**1**](PF_6_)_4_ (1.73 × 10^−5^ M), and [**2**](SbF_6_)_6_ (1.38 × 10^−5^ M) in DMF in a 1 cm thick glass cell. Fig. S36: normalized solid UV-Vis absorption spectra of the KBr-based thin films of **A**, [**1**](BF_4_)_4_, [**1**](ClO_4_)_4_, [**1**](PF_6_)_4_, and [**2**](SbF_6_)_6_ and the pure KBr thin film. Fig. S37: normalized Z-scan data of pure DMF under open-aperture conditions, displaying no detectable signal for absorption performance. Fig. S38: normalized Z-scan data of the pure KBr thin film under open-aperture conditions, displaying no detectable signal for absorption performance. Fig. S39: normalized Z-scan data of **A** in DMF (1.38 × 10^−4^ mol/L) under open-aperture conditions, displaying no detectable signal for absorption performance. Fig. S40: normalized Z-scan data of the **A**/KBr thin film under open-aperture conditions, displaying no detectable signal for absorption performance. Fig. S41: normalized Z-scan data of the KBr-based thin film of [**1**](ClO_4_)_4_ and its DMF solution under open-aperture conditions, displaying a reversed saturable absorption response. Fig. S42: normalized Z-scan data of the KBr-based thin film of [**1**](PF_6_)_4_ and its DMF solution under open-aperture conditions, displaying a reversed saturable absorption response. Fig. S43: normalized Z-scan data of DMF solutions of [**1**](BF_4_)_4_ at concentrations 2.07 × 10^−4^ mol/L and 2.76 × 10^−4^ mol/L under open-aperture conditions. Fig. S44: normalized Z-scan data of DMF solutions of [**2**](SbF_6_)_6_ at concentrations 2.07 × 10^−4^ mol/**L** and 2.76 × 10^−4^ mol/L under open-aperture conditions. Table S1: summary of crystal data and structure refinement parameters for [**1**](BF_4_)_4_, [**1**](ClO_4_)_4_, [**1**](PF_6_)_4_, and [**2**](SbF_6_)_6_. Table S2: the third-order NLO parameters for [**1**](BF_4_)_4_ in DMF, [**1**](ClO_4_)_4_ in DMF, [**1**](PF_6_)_4_ in DMF, [**2**](SbF_6_)_6_ in DMF, [**1**](BF_4_)_4_/KBr thin film, [**1**](ClO_4_)_4_/KBr thin film, [**1**](PF_6_)_4_/KBr thin film, and [**2**](SbF_6_)_6_/KBr thin film.
