# Supplementary material for: Flexible Vertex Engineers the Controlled Assembly of Distorted Supramolecular Tetrahedral and Octahedral Cages
Source: Research (Wash D C). 2022 Feb 24;2022:9819343. doi: 10.34133/2022/9819343 (PMC8897743; doi:10.34133/2022/9819343)
Supplement: Supplementary Materials — Fig. S1: experimental (top) and theoretical (bottom) ESI-TOF-MS spectra of {[1](BF4)}3+ in [1](BF4)4. Fig. S2: experimental (top) and theoretical (bottom) ESI-TOF-MS spectra of {[1](ClO4)}3+ in [1](ClO4)4. Fig. S3: experimental (top) and theoretical (bottom) ESI-TOF-MS spectra of {[1](PF6)}3+ in [1](PF6)4. Fig. S4: experimental (top) and theoretical (bottom) ESI-TOF-MS spectra of {[1](PF6)2}2+ in [1](PF6)4. Fig. S5: experimental (top) and theoretical (bottom) ESI-TOF-MS spectra of {[2](SbF6)(Cl)·10H2O}4+ in [2](SbF6)6. Fig. S6: 1H NMR spectrum of ligand L (400 MHz, 298 K, CDCl3, ppm). Fig. S7: 13C NMR spectrum of ligand L (101 MHz, 298 K, CDCl3, ppm). Fig. S8: 1H NMR spectrum of [1](BF4)4 (600 MHz, 298 K, CD3CN, ppm). Fig. S9: 1H-1H COSY NMR spectrum of [1](BF4)4 (600 MHz, 298 K, CD3CN, ppm). Fig. S10: 1H DOSY NMR spectrum of [1](BF4)4 (600 MHz, 298 K, CD3CN, ppm). Fig. S11: 1H NMR spectrum of [1](ClO4)4 (600 MHz, 298 K, CD3CN, ppm). Fig. S12: 1H-1H COSY NMR spectrum of [1](ClO4)4 (600 MHz, 298 K, CD3CN, ppm). Fig. S13: 1H DOSY NMR spectrum of [1](ClO4)4 (600 MHz, 298 K, CD3CN, ppm). Fig. S14: 1H NMR spectrum of [1](PF6)4 (600 MHz, 298 K, CD3CN, ppm). Fig. S15: 1H-1H COSY NMR spectrum of [1](PF6)4 (600 MHz, 298 K, CD3CN, ppm). Fig. S16: 1H DOSY NMR spectrum of [1](PF6)4 (600 MHz, 298 K, CD3CN, ppm). Fig. S17: 1H NMR spectrum of [2](SbF6)6 (600 MHz, 298 K, CD3CN, ppm). Fig. S18: 1H-1H COSY NMR spectrum of [2](SbF6)6 (600 MHz, 298 K, CD3CN, ppm). Fig. S19: 1H DOSY NMR spectrum of [2](SbF6)6 (600 MHz, 298 K, CD3CN, ppm). Fig. S20: a representation of single vertex (the [Tp∗WS3Cu2(La)]+ unit) consisting of the C3-symmetry Tp∗WS3 moiety, two Cu(I) ions and one linker La in tetrahedral cage [1]. Fig. S21: view of the structure of the tetrahedral cage [1](BF4)4 showing the inclusion of one BF4– with a space filling representation within the cavity as well as three external BF4– anions. Fig. S22: one pair of enantiomers of the tetrahedral cage of [1]. Fig. S23: one pair of [file 9819343.f1.docx]

**Supplementary** **Materials**

**Flexible vertex engineers the controlled assembly of distorted supramolecular tetrahedral and octahedral cages**

Shu-Jin Bao^1,2^, Ze-Ming Xu^1^, Tian-Chen Yu^3^, Ying-Lin Song^3^, Heng Wang^4^, Zheng Niu^1^*, Xiaopeng Li^4^, Brendan F. Abrahams^5^, Pierre Braunstein^6^, Jian-Ping Lang^1,2,^*

^1^College of Chemistry, Chemical Engineering and Materials Science, Soochow University, Suzhou 215123, P. R. China

^2^State Key Laboratory of Organometallic Chemistry, Shanghai Institute of Organic Chemistry, Chinese Academy of Sciences, Shanghai 200032, P. R. China

^3^School of Physical Science and Technology, Soochow University, Suzhou 215006, P. R. China

^4^College of Chemistry and Environmental Engineering, Shenzhen University, Shenzhen 518071, P. R. China

^5^School of Chemistry, University of Melbourne, Victoria 3010, Australia

^6^Université de Strasbourg - CNRS, Institut de Chimie (UMR 7177 CNRS), 4 rue Blaise Pascal-CS 90032, 67081 Strasbourg, France

*Correspondence should be addressed to Jian-Ping Lang; jplang@suda.edu.cn and Zheng Niu; zhengniu@suda.edu.cn

**Table of Contents**

[**Experimental Details** S3](#_Toc93302106)

[**ESI-TOF MS Spectra** S7](#_Toc93302107)

[**NMR Spectra** S10](#_Toc93302108)

[**Absorption Spectra** S25](#_Toc93302109)

[**Third-Order NLO Properties** S26](#_Toc93302110)

[**References** S32](#_Toc93302111)

**Experimental Details**

**General Methods and Procedures**

The starting materials [Et_4_N][Tp*WS_3_] (**A**) (Tp* = hydridotris(3,5-dimethylpyrazol-1-yl)borate) [S1] and the ligand 1,4-di(pyridin-4-yl)buta-1,3-diyne (**L**) [S2] were prepared according to the literature methods. Other starting materials, solvents and reagents were purchased from commercial sources and used as supplied without additional purification unless otherwise mentioned. Elemental analyses (C, H and N) were performed on a Carlo-Erba CHNO-S microanalyzer. Fourier-transform infrared (IR) spectra of the solid samples (KBr tablets) in the range 400-4000 cm^−1^ were recorded on a Varian 1000 spectrometer. Thermogravimetric analyses (TGA) were performed on a Mettler Toledo Star System under a nitrogen atmosphere at a heating rate of 10 °C min^−1^. UV−Vis spectra were recorded on a Varian Cary-50 UV−Vis spectrophotometer. The solid samples used for elemental analysis and TGA were dried overnight at 80 °C in a vacuum oven for removing solvent molecules. ^1^H NMR spectra and ^13^C NMR spectra of the ligand **L** were recorded on BRUKER AVANCE III HD (400 MHz) at room temperature and referenced to the residual protonated solvent for NMR spectra. ^1^H NMR spectra, ^1^H-^1^H COSY spectra and ^1^H DOSY spectra of [**1**](BF_4_)_4_, [**1**](ClO_4_)_4_, [**1**](PF_6_)_4_ and [**2**](SbF_6_)_6_ were recorded on Varian UNITY plus-600 spectrometer at room temperature and referenced to the residual protonated solvent for NMR spectra. Proton chemical shift *δ* H = 7.26 (CDCl_3_) and *δ* H = 1.94 (CD_3_CN) were reported relative to the solvent residual peak. ESI-TOF MS spectra of [**1**](BF_4_)_4_, [**1**](ClO_4_)_4_, [**1**](PF_6_)_4_ and [**2**](SbF_6_)_6_ were recorded on a Bruker micrOTOF-Q III mass spectrometer.

**Synthesis**

Synthesis of 1,4-di(pyridin-4-yl)buta-1,3-diyne (**L**)

According to the literature procedure [S2], ligand **L** was synthesized as follows. A suspension of copper(I) iodide (250 mg, 1.25 mmol), nickel(II) chloride hexahydrate (300 mg, 1.25 mmol) and tetramethylethylenediamine (TMEDA) (0.75 mL, 5.0 mmol) in 120 mL of anhydrous THF was stirred under an inert atmosphere (N_2_) for 10 min. Then, 4-ethynylpyridine (5000 mg, 50.0 mmol) was added, and the mixture was stirred at room temperature over a period of 4 h while air was bubbled through the mixture. After evaporation of the solvent the resulting residue was chromatographed on silica gel (petroleum ether: ethyl acetate = 10:1) to give a colorless solid **L**: 204 mg (99%). ^1^H NMR (400 MHz, CDCl_3_, ppm): *δ* 8.64 (dd, *J* = 4.4 Hz, *J* = 1.6 Hz, 4H), 7.38 (dd, *J* = 4.4 Hz, *J* = 1.6 Hz, 4H). ^13^C NMR (101 MHz, CDCl_3_, ppm): *δ* 150.26, 129.72, 126.37, 80.51, 77.49.

Synthesis of [Tp*WS_3_Cu_2_(**L^a^**)]_4_(BF_4_)_4_ ([**1**](BF_4_)_4_)

A CH_2_Cl_2_/MeCN (40 mL/10 mL) solution of [Et_4_N][Tp*WS_3_] (**A**) (0.071 g, 0.10 mmol), [Cu(MeCN)_4_]BF_4_ (0.063 g, 0.20 mmol) and **L** (0.020 g, 0.10 mmol) was stirred for 6 h under ambient temperature. Then the solution was filtered, and diethyl ether was carefully layered onto the filtrate to generate red crystals in about one week. Red crystals of [**1**](BF_4_)_4_ were isolated, washed with diethyl ether and dried in vacuo. Yield: 0.085 g (85%). ^1^H NMR (600 MHz, CD_3_CN-*d*_3_): *δ* 8.91 – 8.73 (m, 6H), 8.50 (dd, *J* = 24 Hz, 2H), 8.05 (s, 8H), 7.79 – 7.48 (m, 16H), 6.30 – 6.18 (m, 8H), 5.81 – 5.74 (m, 4H), 3.10 – 2.67 (m, 48H), 2.32 – 2.29 (m, 12H), 1.86 – 1.83 (m, 12H). IR (KBr disk): 3439 (vs), 2967 (w), 2925 (w), 2557 (w), 2184 (w), 1607 (s), 1544 (s), 1495 (w), 1450 (m), 1416 (s), 1384 (w), 1355 (s), 1214 (m), 1124 (m), 1084 (vs), 1066 (s), 857 (w), 824 (w), 693 (w), 644 (w), 544 (w) cm^-1^. ESI-MS: *m/z* = 1239.9343 (calcd for {[**1**](BF_4_)}^3+^ 1240.3300). Elemental analysis calcd (%) for C_116_H_120_B_8_Cu_8_F_16_N_32_S_12_W_4_: C 34.99, H 3.04, N 11.26; found: C 34.75, H 2.79, N 11.39.

Synthesis of [Tp*WS_3_Cu_2_(**L^a^**)]_4_(ClO_4_)_4_ ([**1**](ClO_4_)_4_)

The synthesis method was consistent with that of [**1**](BF_4_)_4_. Compound [**1**](ClO_4_)_4_ was obtained by substituting [Cu(MeCN)_4_]BF_4_ (0.063 g, 0.20 mmol) for [Cu(MeCN)_4_]ClO_4_ (0.066 g, 0.20 mmol). Red crystals of [**1**](ClO_4_)_4_ were isolated, washed with diethyl ether and dried in vacuo. Yield: 0.084 g (0.021 mmol, 83%). ^1^H NMR (600 MHz, CD_3_CN-*d*_3_, ppm): *δ* 8.72 (s, 8H), 8.09 (s, 8H), 7.65 (s, 16H), 6.31 – 6.20 (m, 8H), 5.77 (s, 4H), 2.81 – 2.78 (m, 24H), 2.71 (s, 24H), 2.31 – 2.28 (m, 24H). IR (KBr disk): 3450 (s), 2966 (w), 2924 (w), 2556 (w), 2182 (w), 1606 (s), 1545 (s), 1495 (w), 1450 (m), 1415 (s), 1383 (w), 1355 (s), 1215 (m), 1121 (vs), 1108 (vs), 1092 (s), 1068 (s), 1039 (s), 857 (w), 825 (w), 794 (w), 636 (w), 623 (w), 543 (w), 475 (w) cm^-1^. ESI-MS: *m/z* = 1244.2350 (calcd for {[**1**](ClO_4_)}^3+^ 1244.3087). Elemental analysis calcd (%) for C_116_H_120_B_4_Cl_4_Cu_8_N_32_O_16_S_12_W_4_: C 34.55, H 3.00, N 11.12; found: C 34.78, H 3.21, N 11.29.

Synthesis of [Tp*WS_3_Cu_2_(**L^a^**)]_4_(PF_6_)_4_ ([**1**](PF_6_)_4_)

The synthesis method was similar to that for [**1**](BF_4_)_4_. Compound [**1**](PF_6_)_4_ was obtained by substituting [Cu(MeCN)_4_]BF_4_ (0.063 g, 0.20 mmol) for [Cu(MeCN)_4_]PF_6_ (0.075 g, 0.20 mmol). Red crystals of [**1**](PF_6_)_4_ were isolated, washed with diethyl ether and dried in vacuo. Yield: 0.084 g (0.020 mmol, 80%). ^1^H NMR (600 MHz, CD_3_CN-*d*_3_, ppm): *δ* 8.70 (d, *J* = 6.0 Hz, 8H), 8.08 (s, 8H), 7.76 – 7.50 (m, 16H), 6.31 – 6.21 (m, 8H), 5.79 (s, 4H), 3.10 (s, 12H), 2.79 (s, 12H), 2.73 (s, 12H), 2.70 (s, 12H), 2.31 (s, 12H), 1.92 (s, 12H). IR (KBr disk): 3446 (s), 2919 (w), 2850 (w), 2556 (w), 2182 (w), 1610 (s), 1545 (s), 1451 (w), 1417 (s), 1384 (w), 1356 (s), 1216 (m), 1066 (m), 1039 (m), 845 (vs), 693 (w), 644 (w), 558 (m) cm^-1^. ESI-MS: *m/z* = 1259.5831 (calcd for {[**1**](PF_6_)}^3+^ 1259.6500), 1961.8897 (calcd for {[**1**](PF_6_)_2_}^2+^ 1961.9609). Elemental analysis calcd (%) for C_116_H_120_B_4_Cu_8_F_24_N_32_P_4_S_12_W_4_: C 33.06, H 2.87, N 10.64; found: C 33.24, H 2.96, N 10.89.

Synthesis of [Tp*WS_3_Cu_2_(**L^a^**)]_6_(SbF_6_)_6_ ([**2**](SbF_6_)_6_)

The synthesis method was similar to that of [**1**](BF_4_)_4_. Compound [**2**](SbF_6_)_6_ was obtained by substituting [Cu(MeCN)_4_]BF_4_ (0.063 g, 0.20 mmol) for [Cu(MeCN)_4_]SbF_6_ (0.093 g, 0.20 mmol). Red crystals of [**2**](SbF_6_)_6_ were isolated, washed with diethyl ether and dried in vacuo. Yield: 0.089 g (0.013 mmol, 78%). ^1^H NMR (600 MHz, CD_3_CN-*d*_3_, ppm): *δ* 8.68 (d, *J* = 6.0 Hz, 12H), 8.09 (s, 12H), 7.79 – 7.69 (m, 12H), 7.62 (d, *J* = 6.0 Hz, 12H), 6.30 (s, 6H), 6.20 (s, 6H), 5.78 (s, 6H), 3.09 (s, 18H), 2.79 (s, 18H), 2.71 (s, 18H), 2.69 (s, 18H), 2.30 (s, 18H), 1.91 (s, 18H). IR (KBr disk): 3444 (vs), 2969 (w), 2926 (w), 2852 (w), 2557 (w), 2182 (w), 1610 (vs), 1545 (vs), 1497 (w), 1450 (m), 1416 (s), 1384 (w), 1356 (s), 1216 (s), 1066 (m), 1039 (m), 984 (w), 857 (w), 826 (m), 791 (w), 693 (w), 659 (s), 620 (m), 548 (w) cm^-1^. ESI-MS: *m/z* = 1475.0350 (calcd for {[**2**](SbF_6_)(Cl)·10H_2_O}^4+^ 1475.4850). Elemental analysis calcd (%) for C_174_H_180_B_6_Cu_12_F_36_N_48_S_18_Sb_6_W_6_: C 30.44, H 2.64, N 9.79; found: C 30.13, H 2.82, N 9.65.

**X-ray Data Collection and Structure Determination**

Single crystals of [**1**](BF_4_)_4_, [**1**](ClO_4_)_4_, [**1**](PF_6_)_4_ and [**2**](SbF_6_)_6_ suitable for X-ray diffraction were obtained directly from the above preparations. Their single crystals were extremely unstable to solvent loss and rapid handling (< 30 s) at Paratone oil on a Cryoloop pin prior to quenching was required to collect data. Even with these measures the diffraction was broad and weak. The single crystals of [**1**](BF_4_)_4_, [**1**](ClO_4_)_4_, [**1**](PF_6_)_4_ and [**2**](SbF_6_)_6_ coated with Paratone oil on a Cryoloop pin were mounted on a Bruker Smart CCD diffractometer with a graphite monochromated Mo Kα radiation (λ = 0.71073 Å). Indexing was performed using Bruker APEX 2 software package (difference vectors method) [S3]. Data integration and reduction were performed using SAINT v8.34A [S4]. Absorption correction was performed by the multi-scan method implemented in SADABS 2014/5 routines (no correction was made for extinction or decay) [S5]. All crystal structures were solved by Direct methods and refined by full-matrix least-squares techniques on *F*o^2^ using the *SHELXL* program [S6] through the *OLEX*2 interface [S7]. Appropriate restraints or constraints or rigid bodies were applied to the geometry and the atomic displacement parameters of the atoms in these compounds. Hydrogen atoms were geometrically calculated and refined as riding atoms unless otherwise noted. A small amount of spatially delocalized electron density in the lattice was found but acceptable refinement results could not be obtained for this electron density. Because of the poor diffraction quality of the crystal, a number of highly disordered solvent molecules could not be restrained properly and were therefore removed using the SQUEEZE route in the Platon program suite [S8].

Details of the refinement are as follows. For [**1**](BF_4_)_4_, there were disordered solvents which could not be restrained properly. Therefore, SQUEEZE algorithm was used to omit them. 113 ISOR and 15 DFIX instructions were used to restrain BF_4_^–^ anions, the linker **L^a^** and Tp* moieties. For [**1**](ClO_4_)_4_, there were disordered solvents which could not be restrained properly. Therefore, SQUEEZE algorithm was used to omit them. Besides, one 3,5-dimethylpyrazol in Tp* was disordered over two positions (0.49/0.51). 142 ISOR, 15 DFIX, 2 DELU and 5 EADP instructions were used to restrain ClO_4_^–^ anions, the linker **L^a^** and Tp* moieties. The poor single-crystal quality of [**1**](ClO_4_)_4_ inevitably led to one B-level mistake in the Checkcif Report (PLAT342_ALERT_3_B Low Bond Precision on C-C Bonds ............... 0.02045 Ang). For [**1**](PF_6_)_4_, there were disordered solvent molecules which could not be restrained properly. Therefore, SQUEEZE algorithm was used to omit them. 21 ISOR and 59 DFIX instructions were used to restrain PF_6_^–^ anions, the linker **L^a^** and Tp* moieties. For [**2**](SbF_6_)_6_, in asymmetric unit of [**2**](SbF_6_)_6_ (Figure S29), there were disordered solvents which could not be restrained properly. Therefore, SQUEEZE algorithm was used to omit them. Three SbF_6_^–^ anions (including the encapsulated SbF_6_^–^) were all modeled with occupancies of 0.33333. 4 ISOR, 1 SIMU, 8 SADI, 3 RIGU and 2 EADP instructions were used to restrain SbF_6_^–^ anions and the linker **L^a^**. The structure graphics shown were made by using the program Materials Studio and CrystalMaker. A summary of the pertinent crystallographic data for [**1**](BF_4_)_4_, [**1**](ClO_4_)_4_, [**1**](PF_6_)_4_ and [**2**](SbF_6_)_6_ were summarized in Table S1. The X-ray crystallographic data reported in this study have been deposited at the Cambridge Crystallographic Data Centre under accession number CCDC: 2058179 ([**1**](BF_4_)_4_), 2058182 ([**1**](ClO_4_)_4_), 2058185 ([**1**](PF_6_)_4_) and 2058178 ([**2**](SbF_6_)_6_). These data can be obtained free of charge from the Cambridge Crystallographic Data Centre via www.ccdc.cam.ac.uk/data_ request/cif. The CIF files of [**1**](BF_4_)_4_, [**1**](ClO_4_)_4_, [**1**](PF_6_)_4_ and [**2**](SbF_6_)_6_ are available at supplementary data.

**Third-Order NLO Measurements of A, [1](BF_4_)_4_/KBr, [1](ClO_4_)_4_/KBr, [1](PF_6_)_4_/KBr and [2](SbF_6_)_6_/KBr Thin Films and Their Solutions**

DMF solutions of **A**, [**1**](BF_4_)_4_, [**1**](ClO_4_)_4_, [**1**](PF_6_)_4_ and [**2**](SbF_6_)_6_ (1.38 × 10^-4^ mol/L) were placed in a 2 mm quartz cuvette. Their solutions were stable in air and to laser irradiation under experimental conditions. In the UV-Vis spectra of these cage compounds, the linear absorptions at 532 nm are weak (Figure S35). This implies that the intensity loss and temperature change by photon absorption for the NLO measurements at 532 nm are negligible. In addition, these compounds were also pressed into transparent sheet (diameter: 13 mm, thickness: 0.12 mm) via the KBr tablet pressing method. The solid UV-Vis absorption spectra for all KBr-based thin films were measured (Figure S36), indicating the linear absorptions at 532 nm are also weak. The nano-second *Z*-scan technique and a linear polarized laser light (*λ* = 532 nm; repetition rate = 10 Hz; width = 4 ns) generated from a frequency-doubled, mode-locked, Q-switched Nd: YAG laser were applied to measure their third-order NLO properties. The test method was the same as previously described [S9]. All samples were mounted on a computer-controlled translation stage that shifted each sample along the z-axis and all of the measurements were conducted at room temperature.

The nonlinear absorption data of **A** in DMF, [**1**](BF_4_)_4_ in DMF, [**1**](ClO_4_)_4_ in DMF, [**1**](PF_6_)_4_ in DMF, [**2**](SbF_6_)_6_ in DMF, **A**/KBr thin film, [**1**](BF_4_)_4_/KBr thin film, [**1**](ClO_4_)_4_/KBr thin film, [**1**](PF_6_)_4_/KBr thin film, and [**2**](SbF_6_)_6_/KBr thin film were collected using the *Z*-scan technique with open-aperture settings [S10]. For the KBr-based thin films, the incident pulse energy of 21 µj was employed. For the solution samples, the higher incident pulse energy of 28 µj was employed. A valley at the zero point indicates the reverse saturable absorption (RSA) response, and the depth of the valley symbolizes the strength of RSA signal. All experimental data are numerically fitted according to Sheik Bahae’s theory [S11], and the obtained effective nonlinear absorption coefficient *β* value are listed in Table S2.

The transmittance of light (*T*) is a function of the sample’s *Z* position (with respect to the focal point at *Z* = 0). The nonlinear absorption and the linear absorption coefficient are determined by the classical formula below:

$T\left( Z \right)=\frac{\alpha_{0}}{\sqrt{\pi}\beta I_{i}\left( Z \right)(1-e^{-\alpha_{0}L})}\int_{-\infty}^{\infty} \ln\left[ 1+\beta I_{i}(Z)\frac{1-e^{-\alpha_{0}L}}{\alpha_{0}}e^{{-\tau}^{2}} \right]d\tau$

where *α*_0_ and *α* (*α* = *β*(*I*_i_)) are the linear and effective third-order NLO absorptive coefficients, τ is the time, and *L* was the sample thickness.

**ESI-TOF MS Spectra**


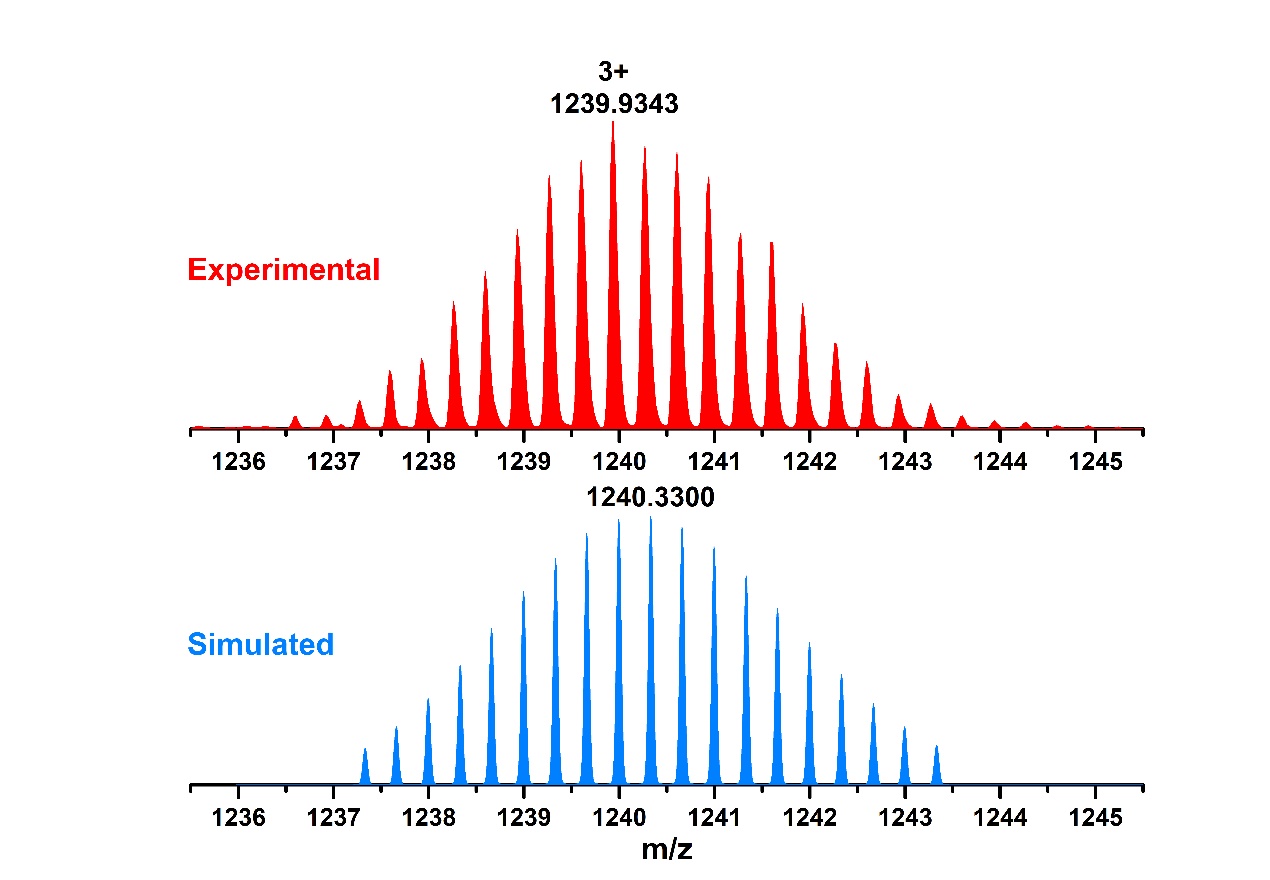


**Figure S1.** Experimental (top) and theoretical (bottom) ESI-TOF-MS spectra of {[**1**](BF_4_)}^3+^ in [**1**](BF_4_)_4_.


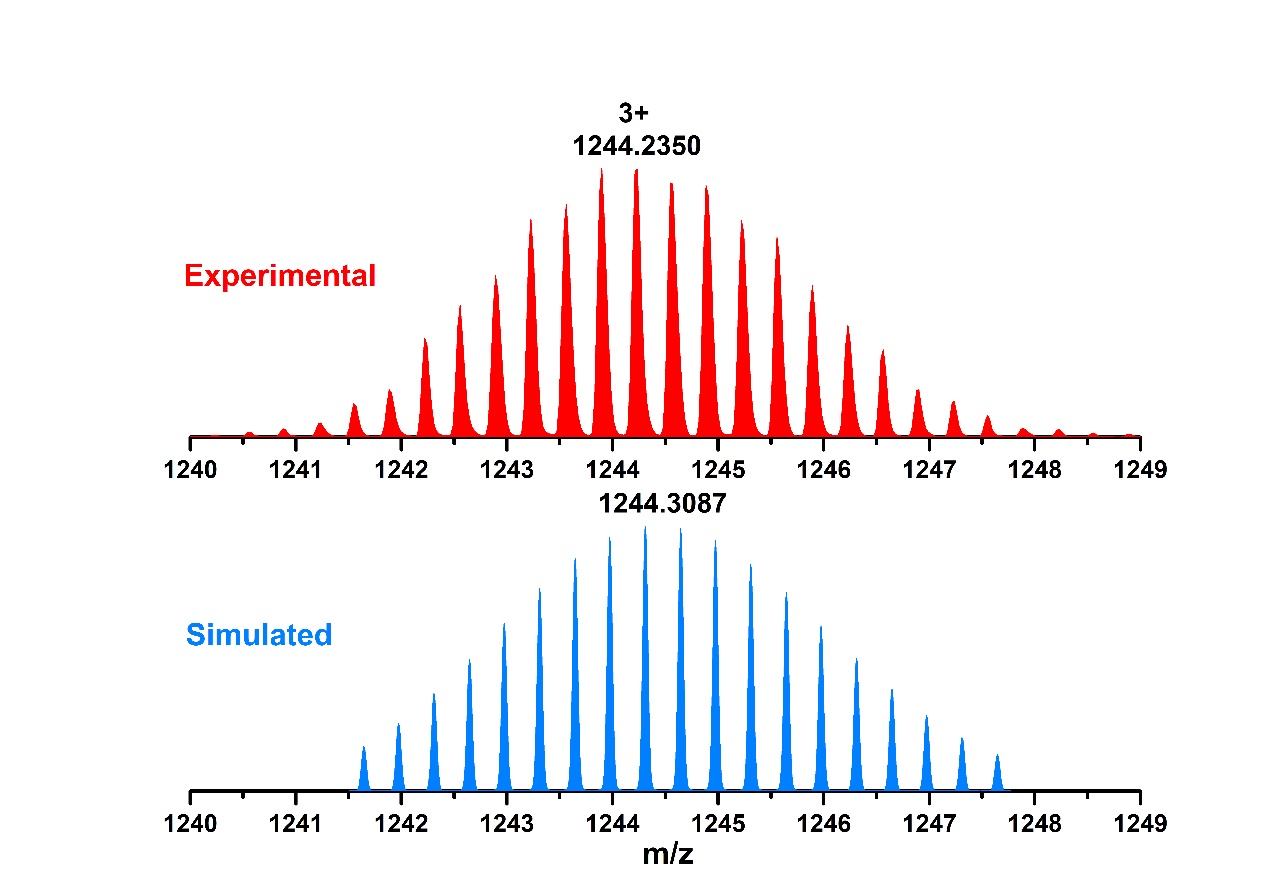


**Figure S2.** Experimental (top) and theoretical (bottom) ESI-TOF-MS spectra of {[**1**](ClO_4_)}^3+^ in [**1**](ClO_4_)_4_.


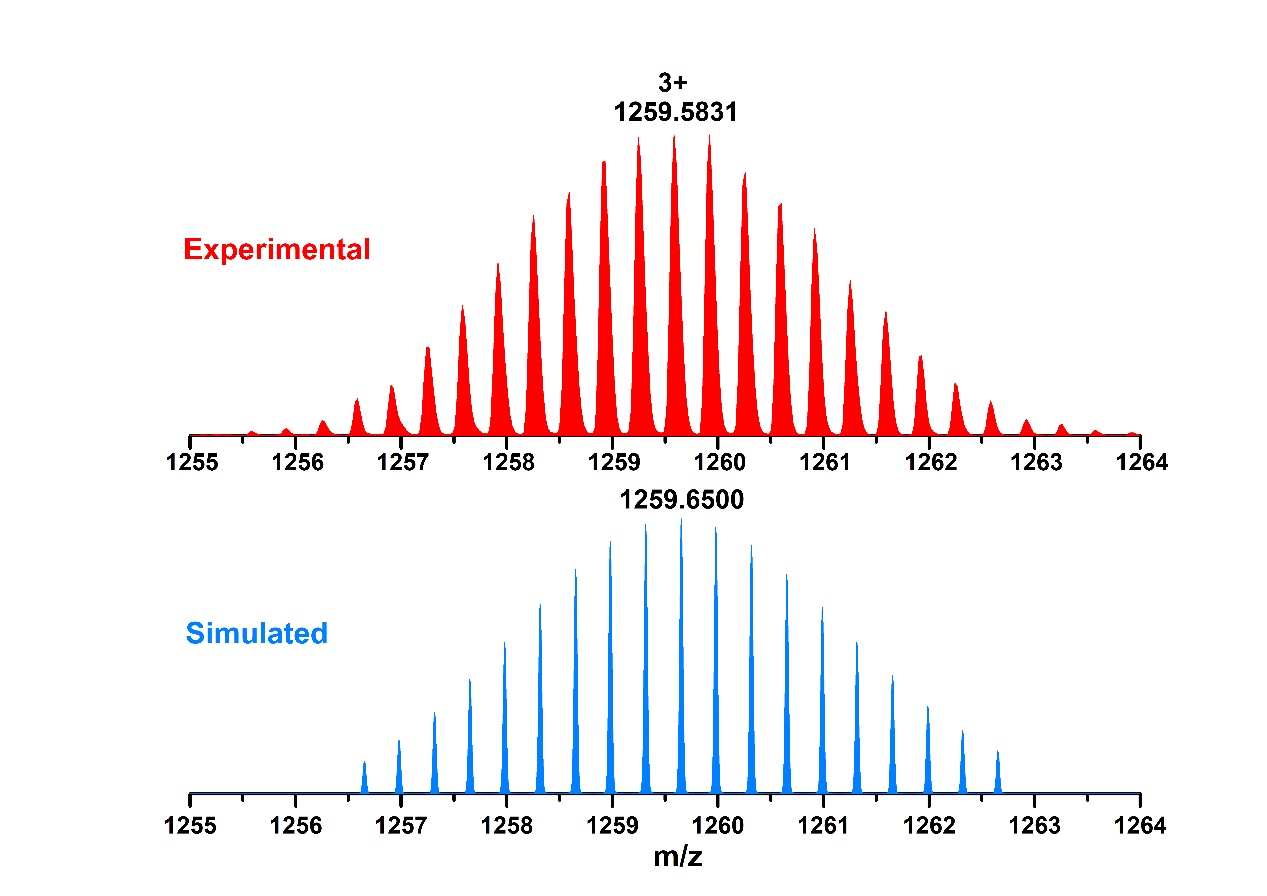


**Figure S3.** Experimental (top) and theoretical (bottom) ESI-TOF-MS spectra of {[**1**](PF_6_)}^3+^ in [**1**](PF_6_)_4_.

**
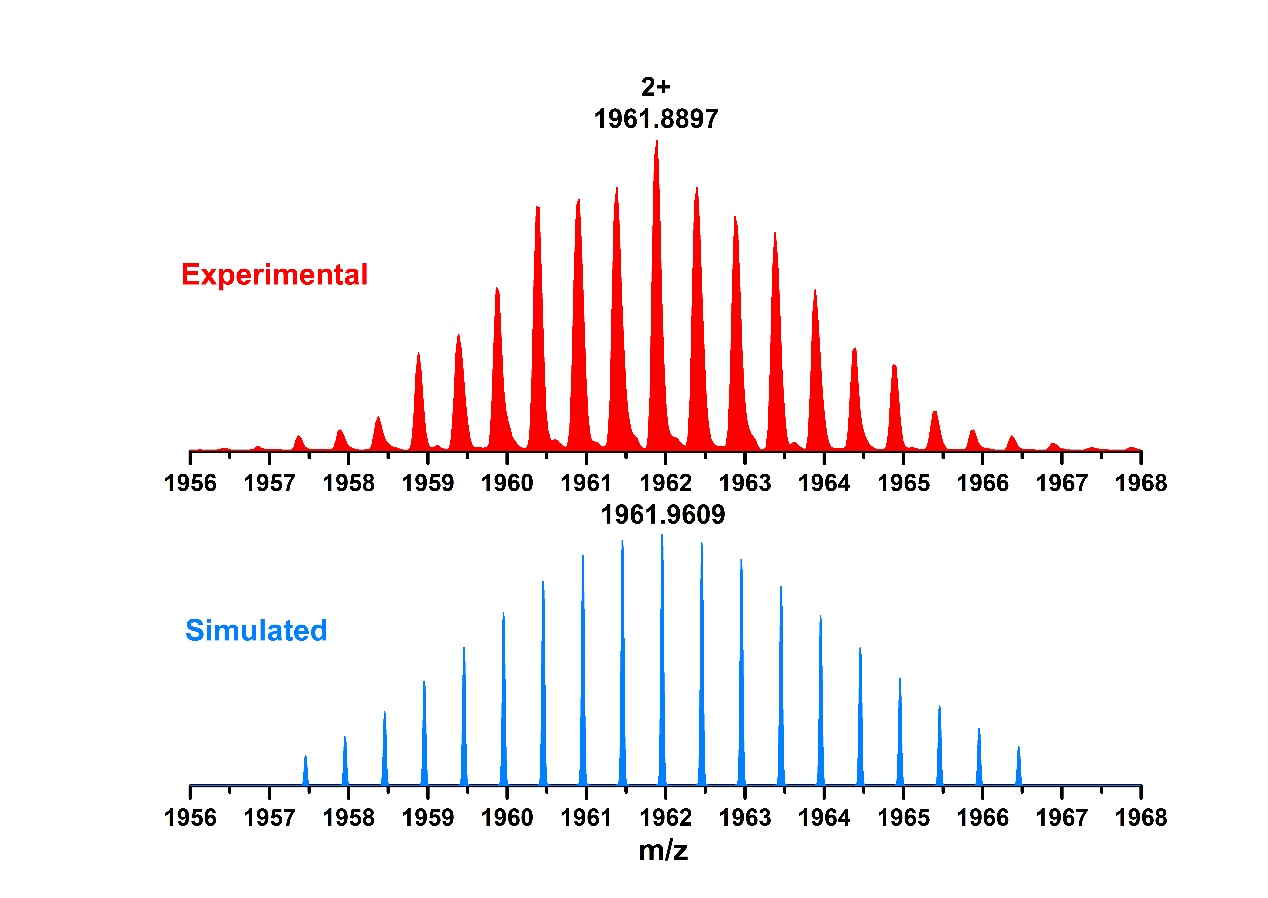
**

**Figure S4.** Experimental (top) and theoretical (bottom) ESI-TOF-MS spectra of {[**1**](PF_6_)_2_}^2+^ in [**1**](PF_6_)_4_.


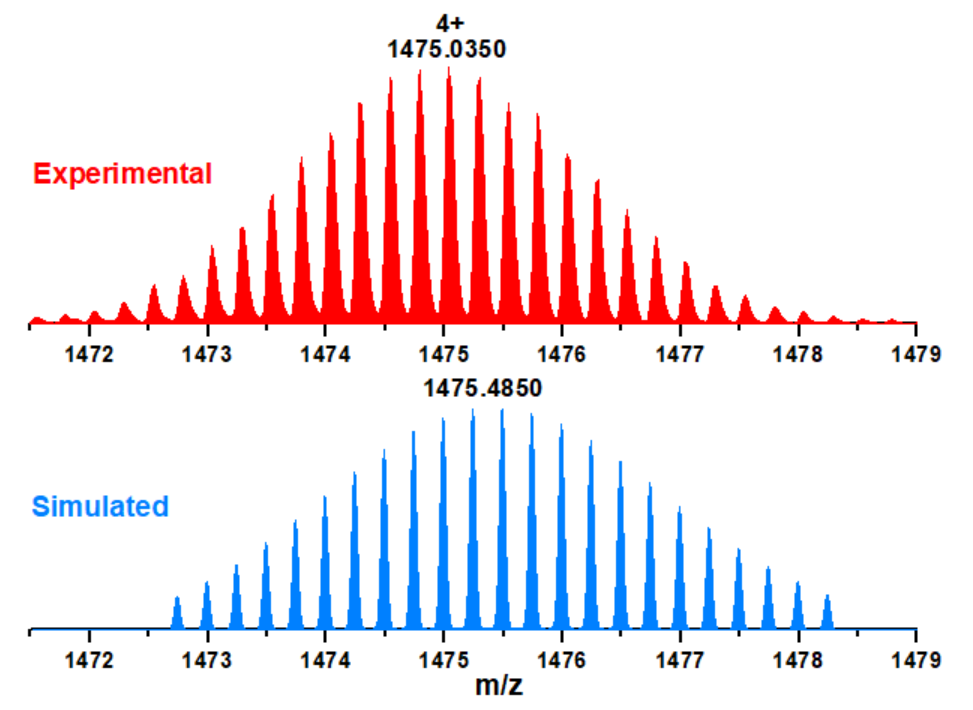


**Figure S5.** Experimental (top) and theoretical (bottom) ESI-TOF-MS spectra of {[**2**](SbF_6_)(Cl)·10H_2_O}^4+^ in [**2**](SbF_6_)_6_.

**NMR Spectra**

**Figure S6.** ^1^H NMR spectrum of ligand **L** (400 MHz, 298 K, CDCl_3_, ppm).

**Figure S7.** ^13^C NMR spectrum of ligand **L** (101 MHz, 298 K, CDCl_3_, ppm).

**Figure S8.** ^1^H NMR spectrum of [**1**](BF_4_)_4_ (600 MHz, 298 K, CD_3_CN, ppm).

**Figure S9.** ^1^H-^1^H COSY NMR spectrum of [**1**](BF_4_)_4_ (600 MHz, 298 K, CD_3_CN, ppm).


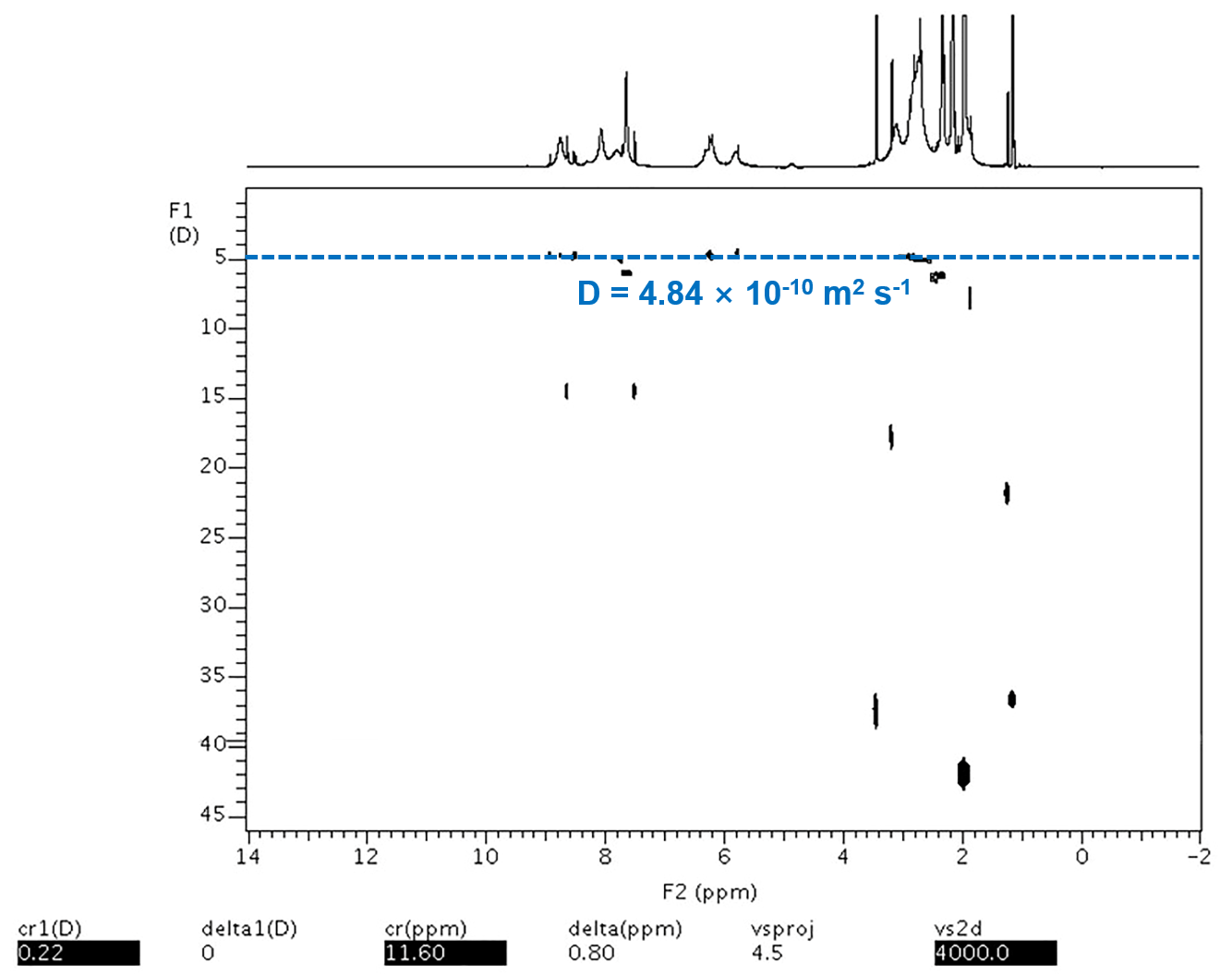


**Figure S10.** ^1^H DOSY NMR spectrum of [**1**](BF_4_)_4_ (600 MHz, 298 K, CD_3_CN, ppm). The diffusion coefficient of [**1**](BF_4_)_4_ in CD_3_CN was measured to be 4.84 × 10^-10^ m^2^ s^-1^.

**Figure S11.** ^1^H NMR spectrum of [**1**](ClO_4_)_4_ (600 MHz, 298 K, CD_3_CN, ppm).

**Figure S12.** ^1^H-^1^H COSY NMR spectrum of [**1**](ClO_4_)_4_ (600 MHz, 298 K, CD_3_CN, ppm).

**Figure S13.** ^1^H DOSY NMR spectrum of [**1**](ClO_4_)_4_ (600 MHz, 298 K, CD_3_CN, ppm). The diffusion coefficient of [**1**](ClO_4_)_4_ in CD_3_CN was measured to be 7.79 × 10^-10^ m^2^ s^-1^.

**Figure S14.** ^1^H NMR spectrum of [**1**](PF_6_)_4_ (600 MHz, 298 K, CD_3_CN, ppm).

**Figure S15.** ^1^H-^1^H COSY NMR spectrum of [**1**](PF_6_)_4_ (600 MHz, 298 K, CD_3_CN, ppm).

**Figure S16.** ^1^H DOSY NMR spectrum of [**1**](PF_6_)_4_ (600 MHz, 298 K, CD_3_CN, ppm). The diffusion coefficient of [**1**](PF_6_)_4_ in CD_3_CN was measured to be 5.81 × 10^-10^ m^2^ s^-1^.

**Figure S17.** ^1^H NMR spectrum of [**2**](SbF_6_)_6_ (600 MHz, 298 K, CD_3_CN, ppm).

**Figure S18.** ^1^H-^1^H COSY NMR spectrum of [**2**](SbF_6_)_6_ (600 MHz, 298 K, CD_3_CN, ppm).

**Figure S19.** ^1^H DOSY NMR spectrum of [**2**](SbF_6_)_6_ (600 MHz, 298 K, CD_3_CN, ppm). The diffusion coefficient of [**2**](SbF_6_)_6_ in CD_3_CN was measured to be 5.03 × 10^-10^ m^2^ s^-1^.

**X-ray Single Crystal Structure**


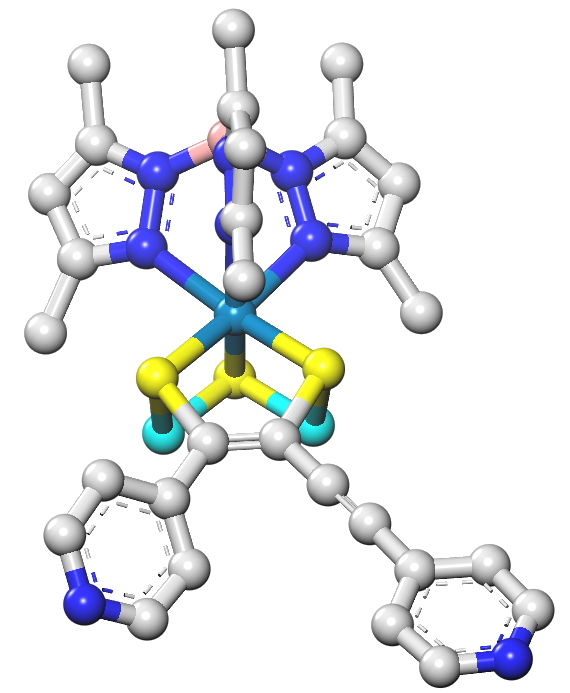


**Figure S20**. A representation of single vertex (the [Tp*WS_3_Cu_2_(**L^a^**)]^+^ unit) consisting of the *C*_3_-symmetry Tp*WS_3_ moiety, two Cu(I) ions and one linker **L^a^** in tetrahedral cage [**1**]. Color codes: W (cyan), Cu (azure), S (yellow), N (blue), C (silver), B (light salmon). All hydrogen atoms, guest solvent and anions have been omitted for clarity.


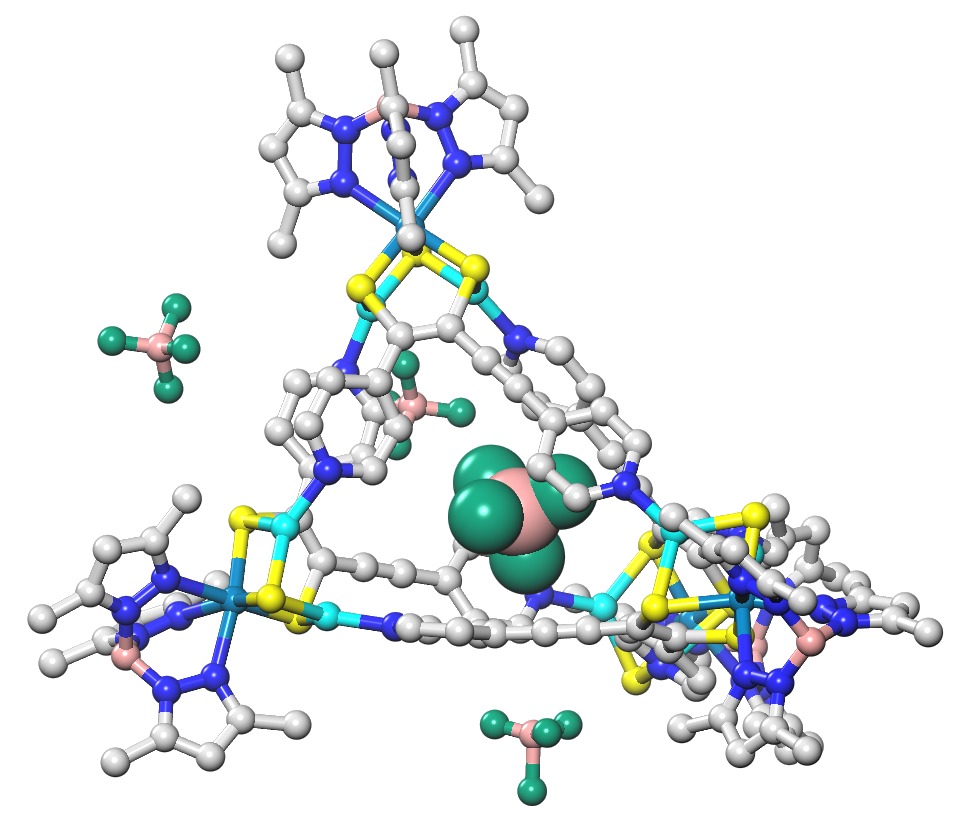


**Figure S21**. View of the structure of the tetrahedral cage [**1**](BF_4_)_4_ showing the inclusion of one BF_4_^–^ with a space filling representation within the cavity as well as three external BF_4_^–^ anions. Color codes: W (cyan), Cu (azure), S (yellow), N (blue), C (silver), B (light salmon), F (dark green). All hydrogen atoms and guest solvent have been omitted for clarity.

**Figure S22**. One pair of enantiomers of the tetrahedral cage of [**1**]. Color codes: W (cyan), Cu (azure), S (yellow), N (blue), C (silver). All hydrogen atoms, guest solvent, anions and the Tp* moieties have been omitted for clarity.


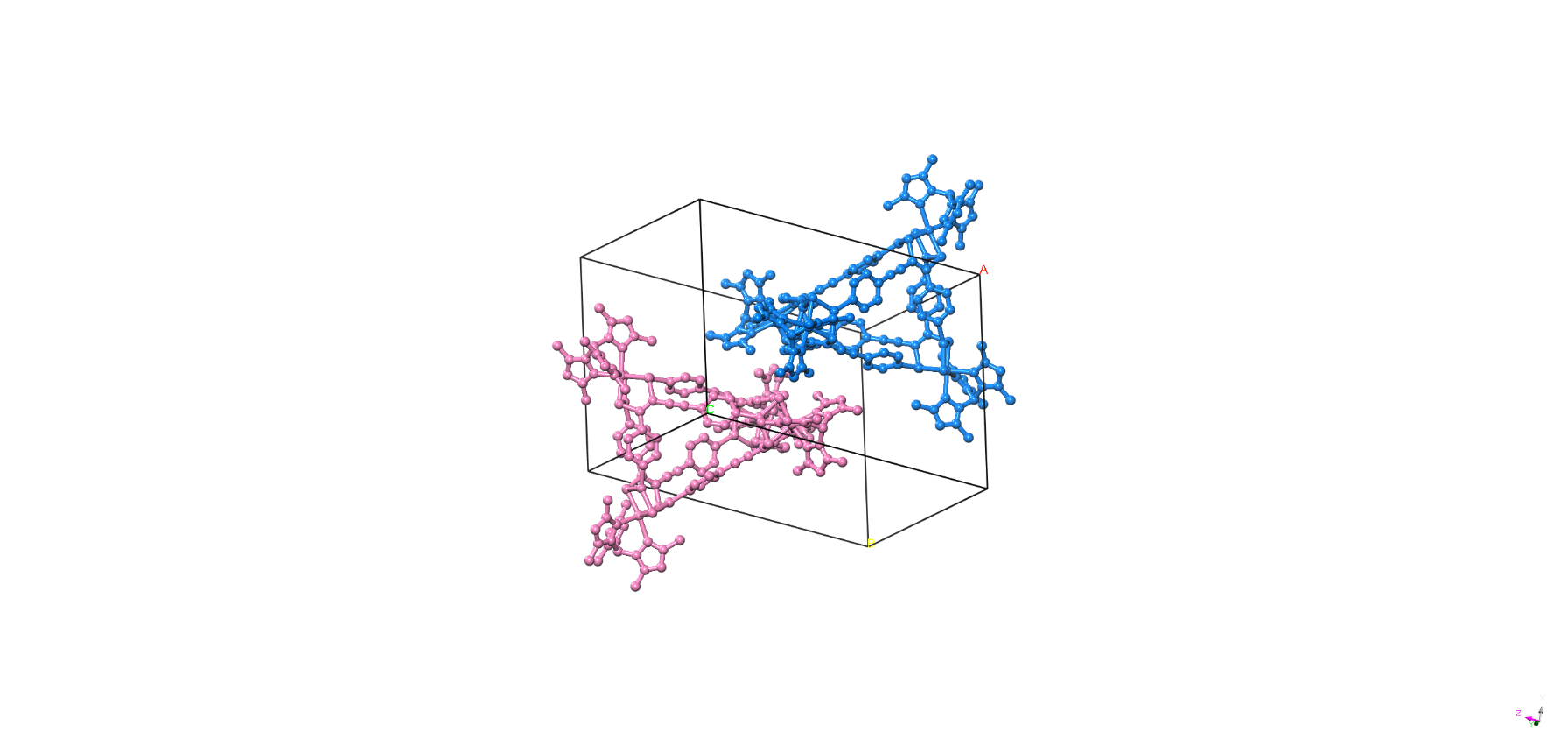


**Figure S23**. One pair of enantiomers packing in one unit cell of [**1**](BF_4_)_4_, forming a racemic crystal. Two enantiomers were represented in pink and blue, respectively. All hydrogen atoms, guest solvent and BF_4_^–^ anions have been omitted for clarity.

**Figure S24**. The host-guest interactions between the internal BF_4_^−^ and the tetrahedral host [**1**] in [**1**](BF_4_)_4_. (A) The hydrogen bonding interactions. (B) The F···π interactions. Color codes: W (cyan), Cu (azure), S (yellow), N (blue), C (silver), B (light salmon), F (dark green), H (light green). The Tp* moieties, guest solvent and free anions have been omitted for clarity.

**Figure S25**. Single-crystal X-ray structure of the tetrahedral cages [**1**](ClO_4_)_4_ and [**1**](PF_6_)_4_. (A) View of the structure of the tetrahedral cage [**1**](ClO_4_)_4_ showing the inclusion of one ClO_4_^–^ with a space filling representation within the cavity as well as three external ClO_4_^–^ anions. (B) View of the structure of the tetrahedral cage [**1**](PF_6_)_4_ showing the inclusion of one PF_6_^–^ with a space filling representation within the cavity as well as three external PF_6_^–^ anions. Color codes: W (cyan), Cu (azure), S (yellow), N (blue), C (silver), B (light salmon), Cl (light green), O (pink), F (dark green), P (magenta). All hydrogen atoms and guest solvent have been omitted for clarity.

**Figure S26**. The topology of three isostructural tetrahedral assemblies [**1**](BF_4_)_4_, [**1**](ClO_4_)_4_ and [**1**](PF_6_)_4_, showing the angles of α in vertex being 42.51°, 42.26° and 43.60°, respectively. The cyan and yellow balls respectively stand for the Tp*WS_3_ unit and reacted alkynyl ligand unit. Color codes: B (light salmon), F (dark green), Cl (light green), O (pink), P (magenta). All hydrogen atoms, guest solvent, free anions and the Tp* moieties have been omitted for clarity.

**Figure S27**. The host-guest interactions between the internal ClO_4_^–^ and the tetrahedral host [**1**] in [**1**](ClO_4_)_4_. (A) The hydrogen bonding interactions. (B) The O···π interactions. Color codes: W (cyan), Cu (azure), S (yellow), N (blue), C (silver), Cl (light green), O (pink), H (red). The Tp* moieties, guest solvent and free anions have been omitted for clarity.

**Figure S28**. The host-guest interactions between the internal PF_6_^−^ and the tetrahedral host [**1**] in [**1**](PF_6_)_4_. (A) The hydrogen bonding interactions. (B) The F···π interactions. Color codes: W (cyan), Cu (azure), S (yellow), N (blue), C (silver), F (dark green), P (magenta), H (light green). The Tp* moieties, guest solvent and free anions have been omitted for clarity.


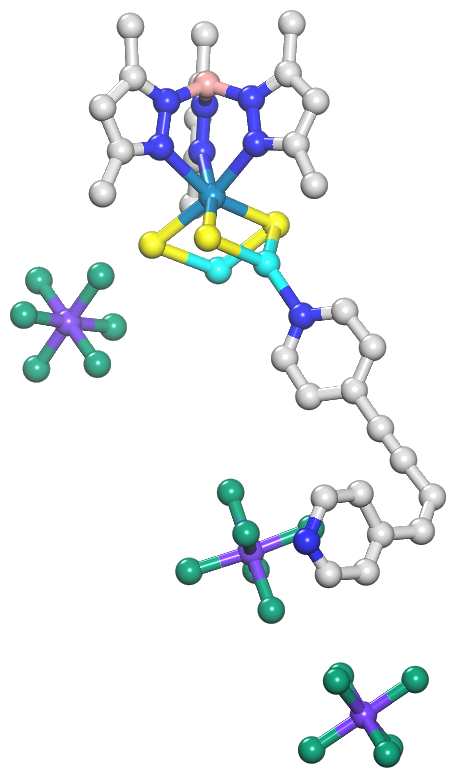


**Figure S29.** View of the asymmetric unit of [**2**](SbF_6_)_6_. Color codes: W (cyan), Cu (azure), S (yellow), N (blue), C (silver), B (light salmon), F (dark green), Sb (purple). All hydrogen atoms, guest solvent , and disorder have been omitted for clarity.

**Figure S30.** Single-crystal X-ray structure of octahedral cage [**2**]. (A) View of the structure of the distorted cationic octahedral cage [**2**] with a space-filling model representation of two encapsulated SbF_6_^−^ anions. (B) View of the space-filling model representation of [**2**]. (C) View of the vertex of [**2**] with a big angle of α being 69.8°. (D) Topology of [**2**]. The angle of α marked here corresponds to the angle of vertex in Figure S30C. The cyan balls and yellow balls respectively stand for the Tp*WS_3_ unit and reacted alkynyl ligand **L^a^** units. Color codes: W (cyan), Cu (azure), S (yellow), N (blue), C (silver), B (light salmon), Sb (violet), F (dark green). In all views, all hydrogen atoms, the non-encapsulated anions, solvent molecules, and disorder are omitted for clarity.

**Figure S31.** The TGA curves of [**1**](BF_4_)_4_, [**1**](ClO_4_)_4_, [**1**](PF_6_)_4_ and [**2**](SbF_6_)_6_ in a N_2_ atmosphere from room temperature to 800 °C.


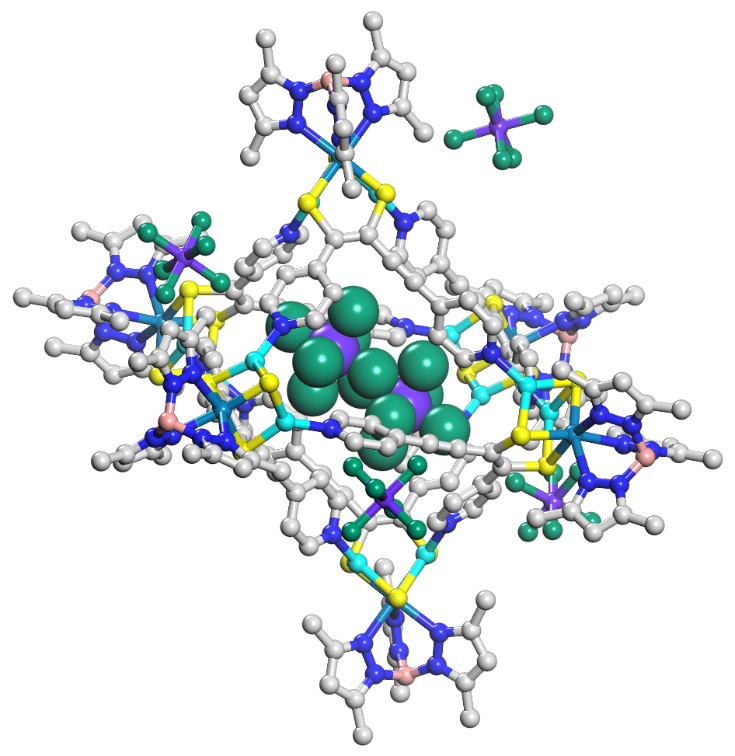


**Figure S32**. View of the structure of the octahedral cage [**2**](SbF_6_)_6_ showing the inclusion of two SbF_6_^–^ anions with a space filling representation within the cavity as well as four external SbF_6_^–^ anions. Color codes: W (cyan), Cu (azure), S (yellow), N (blue), C (silver), B (light salmon), F (dark green), Sb (purple). All hydrogen atoms, guest solvent, and disorder have been omitted for clarity.

**Figure S33**. The host-guest interactions between the internal SbF_6_^–^ and the octahedral host [**2**] in [**2**](SbF_6_)_6_. (A) The hydrogen bonding interactions. (B) The F···π interactions. Color codes: W (cyan), Cu (azure), S (yellow), N (blue), C (silver), F (dark green), Sb (purple), H (light green). The Tp* moieties, guest solvent, free anions, and disorder have been omitted for clarity.


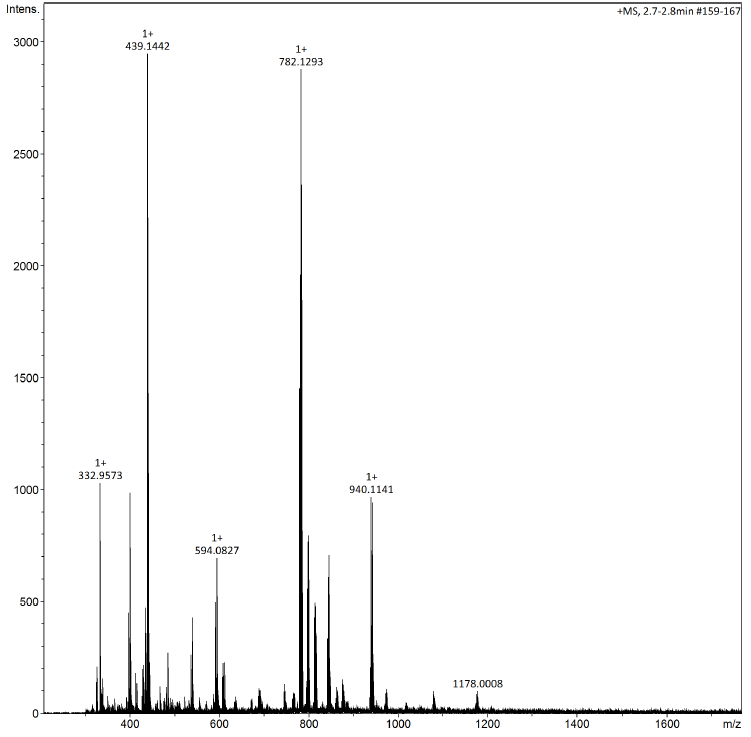


**Figure S34.** The ESI MS spectrum after addition of excess (Et_4_N)(SbF_6_) to the MeCN solution of the tetrahedral compound [**1**](BF_4_)_4_, showing no any octahedral cage signal.

**Absorption Spectra**


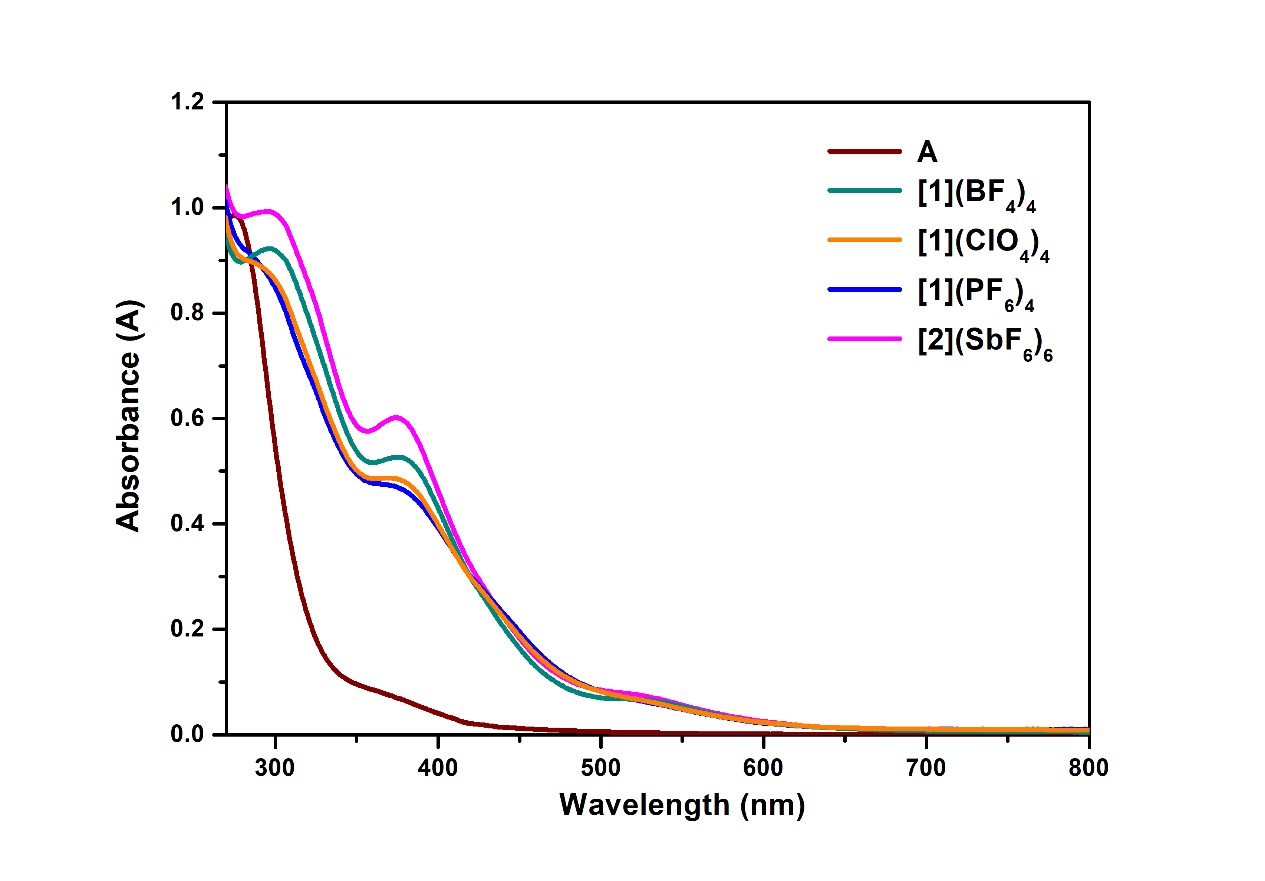


**Figure S35.** Normalized UV-Vis absorption spectra of **A** (8.71 × 10^-5^ M), [**1**](BF_4_)_4_ (1.53 × 10^-5^ M), [**1**](ClO_4_)_4_ (1.73 × 10^-5^ M), [**1**](PF_6_)_4_ (1.73 × 10^-5^ M), and [**2**](SbF_6_)_6_ (1.38 × 10^-5^ M) in DMF in a 1 cm thick glass cell.


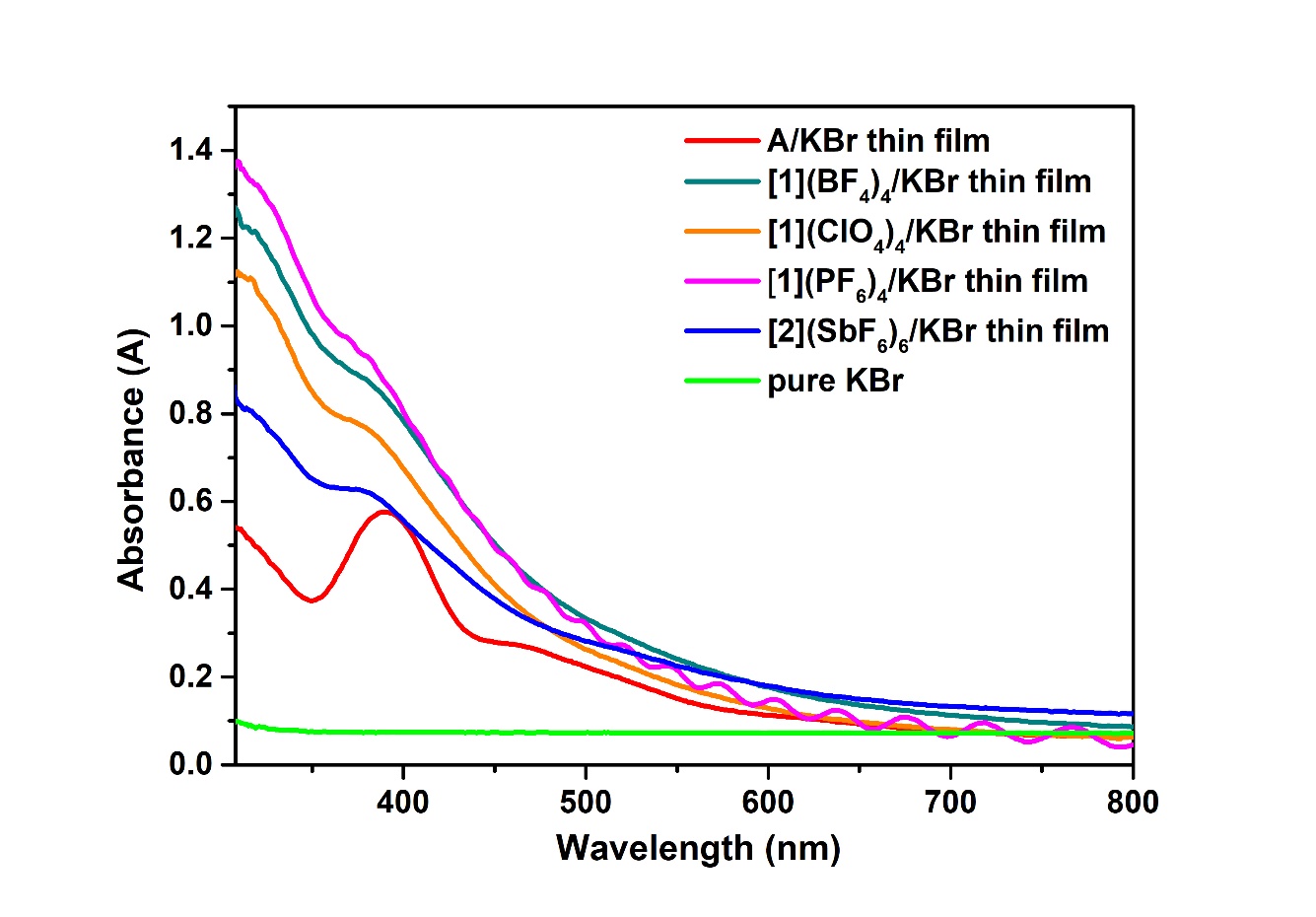


**Figure S36.** Normalized solid UV-Vis absorption spectra of the KBr-based thin films of **A**, [**1**](BF_4_)_4_, [**1**](ClO_4_)_4_, [**1**](PF_6_)_4_ and [**2**](SbF_6_)_6_, and the pure KBr thin film.

**Third-Order NLO Properties**


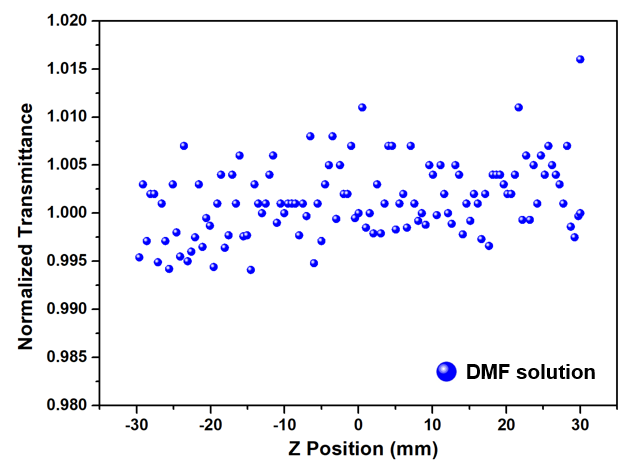


**Figure S37.** Normalized *Z*-scan data of pure DMF under open-aperture conditions, displaying no detectable signal for absorption performance.


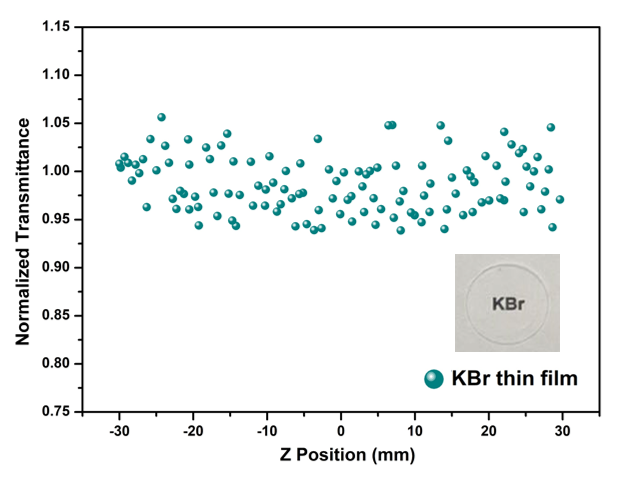


**Figure S38.** Normalized *Z*-scan data of the pure KBr thin film under open-aperture conditions, displaying no detectable signal for absorption performance.

**Figure S39.** Normalized *Z*-scan data of **A** in DMF (1.38 × 10^-4^ mol/L) under open-aperture conditions, displaying no detectable signal for absorption performance.

**Figure S40.** Normalized *Z*-scan data of the **A**/KBr thin film under open-aperture conditions, displaying no detectable signal for absorption performance.

**Figure S41.** Normalized *Z*-scan data of the KBr-based thin film of [**1**](ClO_4_)_4_ and its DMF solution under open-aperture conditions, displaying a reversed saturable absorption response. The violet and wine spheres represent the experimental data of [**1**](ClO_4_)_4_ in DMF and [**1**](ClO_4_)_4_/KBr thin film, respectively, and the green solid curves represent the theoretical fit by Sheik Bahae’s theory.

**Figure S42.** Normalized *Z*-scan data of the KBr-based thin film of [**1**](PF_6_)_4_ and its DMF solution under open-aperture conditions, displaying a reversed saturable absorption response. The blue and pink spheres represent the experimental data of [**1**](PF_6_)_4_ in DMF and [**1**](PF_6_)_4_/KBr thin film, respectively, and the green solid curves represent the theoretical fit by Sheik Bahae’s theory.

**Figure S43.** Normalized *Z*-scan data of DMF solutions of [**1**](BF_4_)_4_ at concentrations 2.07 × 10^-4^ mol/L and 2.76 × 10^-4^ mol/L under open-aperture conditions. The pink and wine spheres represent the experimental data of [**1**](BF_4_)_4_ in DMF (2.07 × 10^-4^ mol/L) and [**1**](BF_4_)_4_ in DMF (2.76 × 10^-4^ mol/L), respectively, and the green solid curves represent the theoretical fit by Sheik Bahae’s theory.

**Figure S44.** Normalized *Z*-scan data of DMF solutions of [**2**](SbF_6_)_6_ at concentrations 2.07 × 10^-4^ mol/L and 2.76 × 10^-4^ mol/L under open-aperture conditions. The blue and purple spheres represent the experimental data of [**2**](SbF_6_)_6_ in DMF (2.07 × 10^-4^ mol/L) and [**2**](SbF_6_)_6_ in DMF (2.76 × 10^-4^ mol/L), respectively, and the green solid curves represent the theoretical fit by Sheik Bahae’s theory.

**Figure S45.** The ESI-TOF MS spectrum of the reaction solution of **A**, [Cu(MeCN)](OTf) and **L**. The two signals at *m/z* = 1119.9339 and 1437.1539 can be assigned to {[2](OTf)}^5+^ and {[2](OTf)_2_}^4+^, respectively.

**Table S1.** Summary of crystal data and structure refinement parameters for [**1**](BF_4_)_4_, [**1**](ClO_4_)_4_, [**1**](PF_6_)_4_ and [**2**](SbF_6_)_6_.

|  | [**1**](BF_4_)_4_ | [**1**](ClO_4_)_4_ | [**1**](PF_6_)_4_ | [**2**](SbF_6_)_6_ |
| --- | --- | --- | --- | --- |
| Empirical formula | C_116_H_120_B_8_Cu_8_F_16_N_32_S_12_W_4_ | C_116_H_120_B_4_Cl_4_Cu_8_N_32_O_16_S_12_W_4_ | C_116_H_120_B_4_Cu_8_F_24_N_32_P_4_S_12_W_4_ | C_174_H_180_B_6_Cu_12_F_36_N_48_S_18_Sb_6_W_6_ |
| Formula weight | 3981.35 | 4031.91 | 4213.99 | 6865.67 |
| Crystal system | triclinic | triclinic | triclinic | trigonal |
| Space group |  | *P* | *P* | *R* |
| *a*/Å | 17.6355(19) | 17.7259(18) | 15.4472(11) | 20.1031(4) |
| *b*/Å | 20.529(2) | 20.846(2) | 18.7854(13) | 20.1031(4) |
| *c*/Å | 24.099(3) | 24.187(2) | 32.958(2) | 71.130(3) |
| *α*/deg | 82.678(3) | 83.556(3) | 96.100(2) | 90 |
| *β*/deg | 78.736(4) | 77.721(3) | 100.493(2) | 90 |
| *γ*/deg | 88.404(4) | 88.834(3) | 90.902(2) | 120 |
| *V*/Å^3^ | 8486.8(16) | 8677.7(15) | 9344.7(11) | 24894.9(13) |
| *ρ*_calc_ g/cm^3^ | 1.558 | 1.543 | 1.498 | 1.374 |
| Z | 2 | 2 | 2 | 3 |
| *μ*/mm^–1^ | 3.892 | 3.861 | 3.578 | 3.471 |
| Reflections collected | 74468 | 95577 | 149159 | 182745 |
| Independent reflections | 38282 | 39405 | 42805 | 12768 |
| F(000) | 3888.0 | 3952.0 | 4112.0 | 9900.0 |
| *R*_1_*^a^* [*I* > 2*α* (*I*)] | 0.0831 | 0.0863 | 0.0750 | 0.0551 |
| *wR*_2_*^b^* | 0.1778 | 0.1994 | 0.1764 | 0.1567 |
| *GOF^c^* | 0.978 | 1.028 | 1.049 | 1.133 |

*^a^* *R* = Σ||*F*_o_|–|*F*_c_||/Σ|*F*_o_|. *^b^* *wR* = {Σ*w*(*F*_o_^2^–*F*_c_^2^)^2^/Σ*w*(*F*_o_^2^)^2^}^1/2^. *^c^* *GOF* = {Σ*w*((*F*_o_^2^–*F*_c_^2^)^2^)]/(*n*–*p*)}^1/2^, where *n* = number of reflections and *p* = total number of parameters refined.

**Table S2.** The third-order NLO parameters for [**1**](BF_4_)_4_ in DMF, [**1**](ClO_4_)_4_ in DMF, [**1**](PF_6_)_4_ in DMF, [**2**](SbF_6_)_6_ in DMF, [**1**](BF_4_)_4_/KBr thin film, [**1**](ClO_4_)_4_/KBr thin film, [**1**](PF_6_)_4_/KBr thin film, and [**2**](SbF_6_)_6_/KBr thin film.

**References**

[S1] H. Seino, Y. Arai, N. Iwata, et al., “Preparation of mononuclear tungsten tris(sulfido) and molybdenum sulfido-tetrasulfido complexes with hydridotris(pyrazolyl)borate coligand and conversion of the former into sulfido-bridged bimetallic complex having Pt(μ-S)_2_WS core,” Inorganic Chemistry, vol. 40, no. 7, pp. 1677–1682, 2001. (DOI: 10.1021/ic0008823)

[S2] M. Albrecht, M. Nieger and A. Schmidt, “A metal-organic framework constructed of 1,4-di(pyridin-4-yl)-buta-1,3-diyne and nickel(II) nitrate,” Zeitschrift für Naturforschung B, vol. 66, no. 2, pp. 209–212, 2011. (DOI: 10.1515/znb-2011-0215)

[S3] *Bruker APEX2*, *v2014.5-0;* Bruker AXS, Inc.: Madison, WI, 2007.

[S4] *SAINT, v8.34A*; Bruker AXS, Inc: Madison, WI, 2013.

[S5] *SADABS*, Bruker AXS, Inc.: Madison, WI, 2014.

[S6] G. M. Sheldrick, “Crystal structure refinement with *SHELXL*,” Acta Crystallographica Section C Structural Chemistry, vol. 71, no. 1, pp. 3–8, 2015. (DOI: 10.1107/S2053229614024218)

[S7] O. V. Dolomanov, L. J. Bourhis, R. J. Gildea, et al., “*OLEX2*: a complete structure solution, refinement and analysis program,” Journal of Applied Crystallography, vol. 42, no. 2, pp. 339–341, 2009. (DOI: 10.1107/S0021889808042726)

[S8] A. L. Spek, “Single-crystal structure validation with the program *PLATON*,” Journal of Applied Crystallography, vol. 36, no. 1, pp. 7–13, 2003. (DOI: 10.1107/S0021889802022112)

[S9] Z. G. Ren, H. X. Li, L. L. Li, et al., “Synthesis, crystal structures and third-order nonlinear optical properties of a new family of double incomplete cubane-like clusters [(η^5^-C_5_Me_5_)_2_Mo_2_(μ_3_-S)_3_SCu_2_X(μ-X)]_2_ (X = Cl^−^, Br^−^, SCN^−^) and cubane-like clusters [(η^5^-C_5_Me_5_)_2_Mo_2_(μ_3_-S)_4_(CuX)_2_] (X = Br^−^, SCN^−^, CN^−^),” Journal of Organometallic Chemistry, vol. 692, no. 11, pp. 2205–2215, 2007. (DOI: 10.1016/j.jorganchem.2007.01.040)

[S10] Z. G. Ren, J. Y. Yang, Y. L. Song, et al., “From *Trans*-[(η^5^-C_5_Me_5_)_2_Mo_2_S_2_(μ-S)_2_] to [(η^5^-C_5_Me_5_)_2_Mo_2_(μ_3_-S)_4_(CuMeCN)_2_]^2+^ to [(η^5^-C_5_Me_5_)_2_Mo_2_(μ_3_-S)_4_Cu_2_]-based polymeric and dimeric clusters: syntheses, structures and enhanced third-order non-linear optical performances,” Dalton Transactions, no. 14, pp. 2578–2587, 2009. (DOI: 10.1039/B819240E)

[S11] M. Sheik-Bahae, A. A. Said, T. H. Wei, et al., “Sensitive measurement of optical nonlinearities using a single beam,” IEEE Journal of Quantum Electronics, vol. 26, no. 4, pp. 760–769, 1990. (DOI: 10.1109/3.53394)
